# Complete genome sequencing of H1N1pdm09 swine influenza isolates from Nigeria reveals likely reverse zoonotic transmission at the human-animal interface in intensive piggery

**DOI:** 10.1080/20008686.2019.1696632

**Published:** 2019-12-02

**Authors:** C.A. Meseko, A. Heidari, G.N. Odaibo, D.O. Olaleye

**Affiliations:** a Regional Center for Animal Influenza, National Veterinary Research Institute, Vom, Nigeria; b Formerly, Istituto Zooprofilattico Sperimentale delle Venezie, (IZSVe), FAO Reference Center for Animal Influenza and Newcastle Disease virus, OIE Reference Laboratory for Avian Influenza and Newcastle Disease virus, OIE Collaborating Laboratory for Diseases at the Human-Animal Interface, Padova, Italy; c WHO National Influenza Center, Department of Virology, College of Medicine, University of Ibadan, Ibadan, Nigeria

**Keywords:** Influenza virus, A/H1N1pdm09, complete genome sequencing, pigs, human-animal interface, Nigeria

## Abstract

Prevailing agro-ecological conditions and intermingling of human and animals in intensive farms in urban and peri-urban areas in Africa favour cross species transmission of pathogens at the human-animal interface. However, molecular epidemiology studies of zoonotic swine influenza viruses in this region are limited. In this study, isolates of pandemic influenza virus (H1N1pdm09) obtained from pigs in Nigeria were fully sequenced. BLAST of swine influenza virus genes from Nigeria was carried out in GenBank and gene alignment was done using MEGA version 7. Maximum likelihood method (PhyML program) was used to determine gene evolutionary relationships with other viruses and phylogenetic trees were constructed to infer genomic clusters and relationship. Swine influenza viruses isolated and sequenced in this study were monophyletic and 99% congenetic with human isolates from Nigeria, Cameroon, Ghana and USA suggesting reverse zoonotic transmission from humans to pigs in intensive husbandry. A Q240R and S31N substitution among others were detected in the haemagglutinin and matrix genes, respectively, indicating potentials for mutations during interspecies co-mingling and transmission. The A/H1N1pdm09 viruses circulating in pigs that are also exposed to avian influenza in the same epidemiological zones could engender emergence of novel viruses with zoonotic or pandemic potential requiring enhanced surveillance and monitoring.

## Introduction

Pigs are mixing vessels of influenza viruses and known reservoirs of virus genes capable of causing emergence of novel influenza virus strains with potential adverse economic and public health consequences [1]. Although the pandemic H1N1pdm09 influenza virus was first reported in human in Mexico in 2009 [2], genetic evidence suggests that it may have circulated undetected for several years in pigs prior to its recognized spillover transmission into the human population [3]. Surveillance for swine influenza in animals have also shown that the human pandemic virus was transmitted to pigs in Canada and USA [4]. It has also been observed that the H1N1pdm09 virus evolves more rapidly in pigs than in humans [5]. Although millions of pigs are reared in close proximity to humans in urban and peri-urban settings in Nigeria, there is a dearth of epidemiologic and genomic data on swine influenza viruses in the country and in sub-Saharan Africa [6].

The pandemic H1N1pdm09 has been shown to be a product of gene reassortment of avian, swine and human influenza viruses [3]. The ecological and biological elements that engendered such a reassortment events are favoured by intensive livestock farming practices and intermingling at the human-animal interface in urban and peri-urban settings. Practices where pigs are reared in large numbers in enclosures with frequent contacts with human handlers (farmers, farm attendants, traders and butchers) predispose to inter and intra-species transmission and exchange of influenza viruses at the human-animal interface thus favouring the emergence of novel strains. Such emerging strains may possess markers of molecular changes in the influenza virus genome that may be important for transmission and pathogenicity of the virus [7]The ecology, transmission and molecular epidemiology of pandemic H1N1pdm09 in sub-Saharan Africa are yet to be fully understood [2:6]. To improve our comprehension of the genomic and epidemiologic dynamics of pandemic swine-origin H1N1pdm09 isolates from Nigeria, we carried out a complete genome sequencing and phylogenetic study of isolates of swine influenza virus obtained from embryonated egg culture of samples collected from pigs with respiratory signs in a peri-urban piggery in Lagos State, Nigeria.

## Material and method

### Sample collection and virus isolation

Influenza virus surveillance at the human-animal interface was carried out between July 2010 and June 2012 in a multi-complex commercial piggery operation located in a peri-urban zone in Lagos, southwestern Nigeria. Samples were collected from 227 pigs showing upper respiratory signs compatible with influenza-like infection and were screened for the presence matrix gene by real-time RT-PCR [8]. The study site, sampling strategy, molecular detection and virus isolation methods and results have been previously described [9]. Out of 227 samples analysed, 31 (13.7%) were positive for influenza A matrix gene by real-time RT-PCR. Virus isolation yielded 29 (12%) isolates out of which 18 (18%) were identified as influenza A/H1N1 by Heamaglutination Inhibition test against H1 antisera.

### Reverse transcription polymerase chain reaction

RT-PCR positive samples were also subtyped as 2009 pandemic A/H1N1 with subtype-specific primers and probes. Each gene segments were further amplified using several PCRs targeting overlapping fragments with cycling conditions set at 95°C for 2 min, 95°C for 45 s, 46°C for 1 min, 72°C for 4 min, 72°C for 10 min and 4°C (hold; infinity) for 40 cycles (PCR primers used for fragment of each gene segments are provided as supplementary material. Following amplification, PCR products were purified using ExoSAP-IT to remove unwanted primers and dNTPS.

### Genomic sequencing and characterization

Complete genome sequencing of all eight gene segments of three viral isolates and partial sequences for 12 RT-PCR positive samples was carried out by Sanger method using the Big Dye Terminator v3.1 (3130x Genetic Analyser, Applied Biosystems, Foster City, CA, USA). (not all isolates obtained in Nigeria were re-isolated at the OIE Reference laboratory in Padova, Italy due to sample deterioration at storage and transportation). Cycle condition for sequence reaction was set at: 96°C for 10 s, 50°C for 5 s and 60°C for 4 min for 25 cycles. This was followed by gel filtration clean up of sequenced products using Column AutoSeq G-50 before analysis in 16 capillary Sequence Analyser (3130x Genetic Analyser, Applied Biosystem, Foster City, CA, USA). Subsequently, sequence assembly and editing were performed using Seqscape version 2.5 (Applied Biosystem, Foster City, CA, USA). Gene sequences were thereafter compared with corresponding genes of other influenza virus strains obtained from GenBank and Global Initiative on Sharing All Influenza Data (GISAID). These include human influenza sequences from Nigeria, Ghana and Cameroon, viz.: A/Nigeria/4280/2011, A/Ghana/601/2011 and A/Cameroon/EID/07/11/1870/2011.

### Phylogenetic analyses

To infer the evolutionary relationship for each gene segment, we employed maximum likelihood (ML) methods available through the PhyML program, incorporating a GTR model of nucleotide substitution with gamma-distributed rate variation among sites and a heuristic SPR branch-swapping search Parameter values for the GTR substitution matrix, base composition, gamma distribution of the rate variation among sites (with four rate categories) and proportion of invariant sites (I) were estimated directly from the data using MEGA 7. A bootstrap resampling process (1,000 replications) using the neighbor-joining (NJ) method and incorporating the ML substitution model defined above, was employed to assess the robustness of individual nodes of the phylogeny [10]. Nucleotides and amino acid substitutions of influenza H1N1pdm09 isolates from Nigeria were compared with prototype A/California/07/2009(H1N1) and phylogenetic trees were constructed for all the genes to assess genomic clusters and relationships. Though three isolates were used to construct the phylogenetic trees, some of the genes only had two clearly resolved sequences used to construct the tree for such gene.

## Results

The pandemic Swine influenza virus that were isolated from the piggery complex in Nigeria and used in the phylogenetic study has earlier been reported [9]. The GenBank basic local alignment search tool (BLAST) of sequenced genes showed that the virus strains with the closest identity to Nigerian isolates with 100% query coverage was A/SanDiego/INS14-49-104, 2009(H1N1) and related sequences with above 99% sequence homology for HA, PB1, PB2, PA and NP genes while NA, MA and NS genes with 100% query coverage showed above 99% homology with A/California/VRDL113/2009(H1N1), A/Malaysia/2,089,302/2009(H1N1) and A/HongKong/H090-693-v20/2009(H1N1), respectively. Phylogenetically, the nucleotide sequences of the H1N1pdm09 isolates from Nigeria were identical and clustered together with global H1N1pdm09 viruses from San Diego, Singapore and Germany (Figure 1) for HA, NA and all the internal genes. The full gene (complete genome) sequences of (A/swine/Nigeria/12VIR4047/2011) has been deposited in the GenBank with accession number JX442481- HA, JX4442482- NA, JX482555-PB2, JX482556-PB1, JX482557-MA, JX482558-NP, JX482559-PA and JX48260-NS (www.ncbi.nlm.nih.gov).

**Figure 1.**
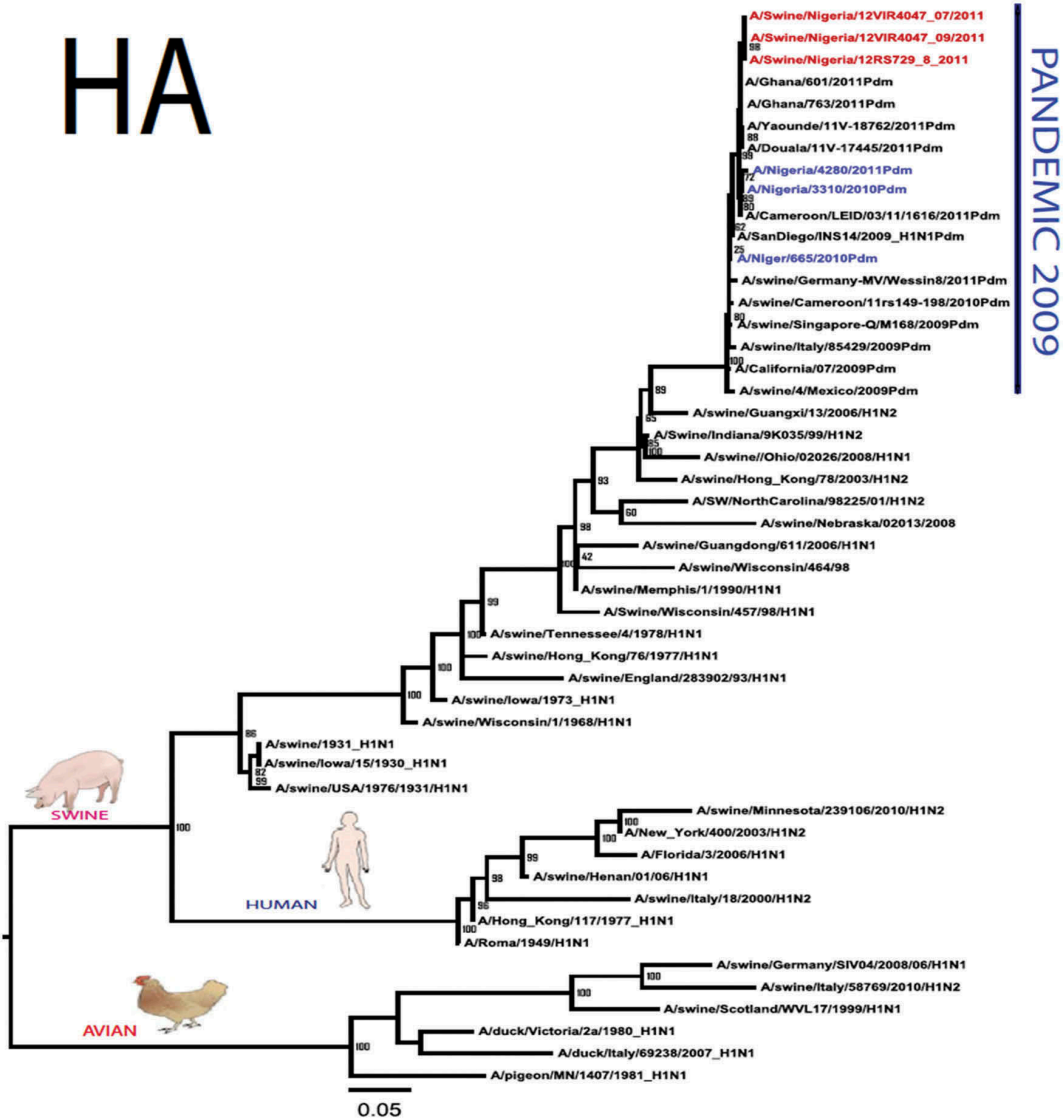
Phylogenetic tree of H1N1pdm09 influenza A virus (A/swine/Nigeria/12VIR4047/2011) isolated in Nigeria (HA, NA, MA, PB1, PB2, PA, NS and NP genes). In red colour are swine isolates from Nigeria while blue colour represents human isolates from Nigeria.

**Figure 1.**
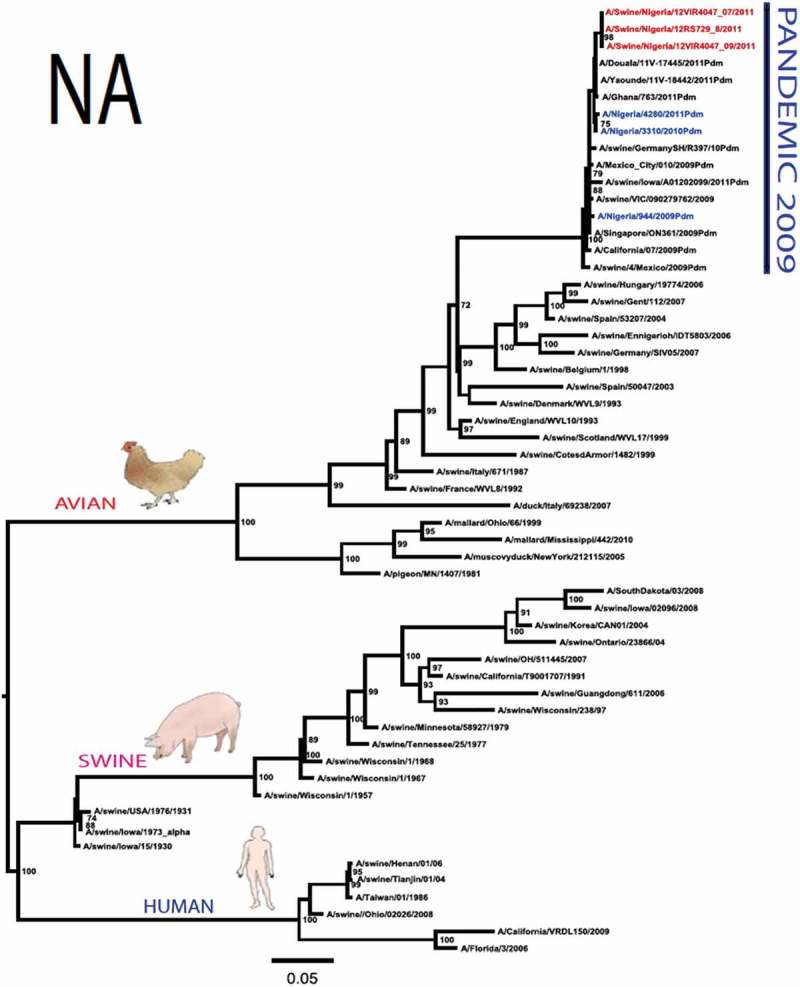
Continued.

**Figure 1.**
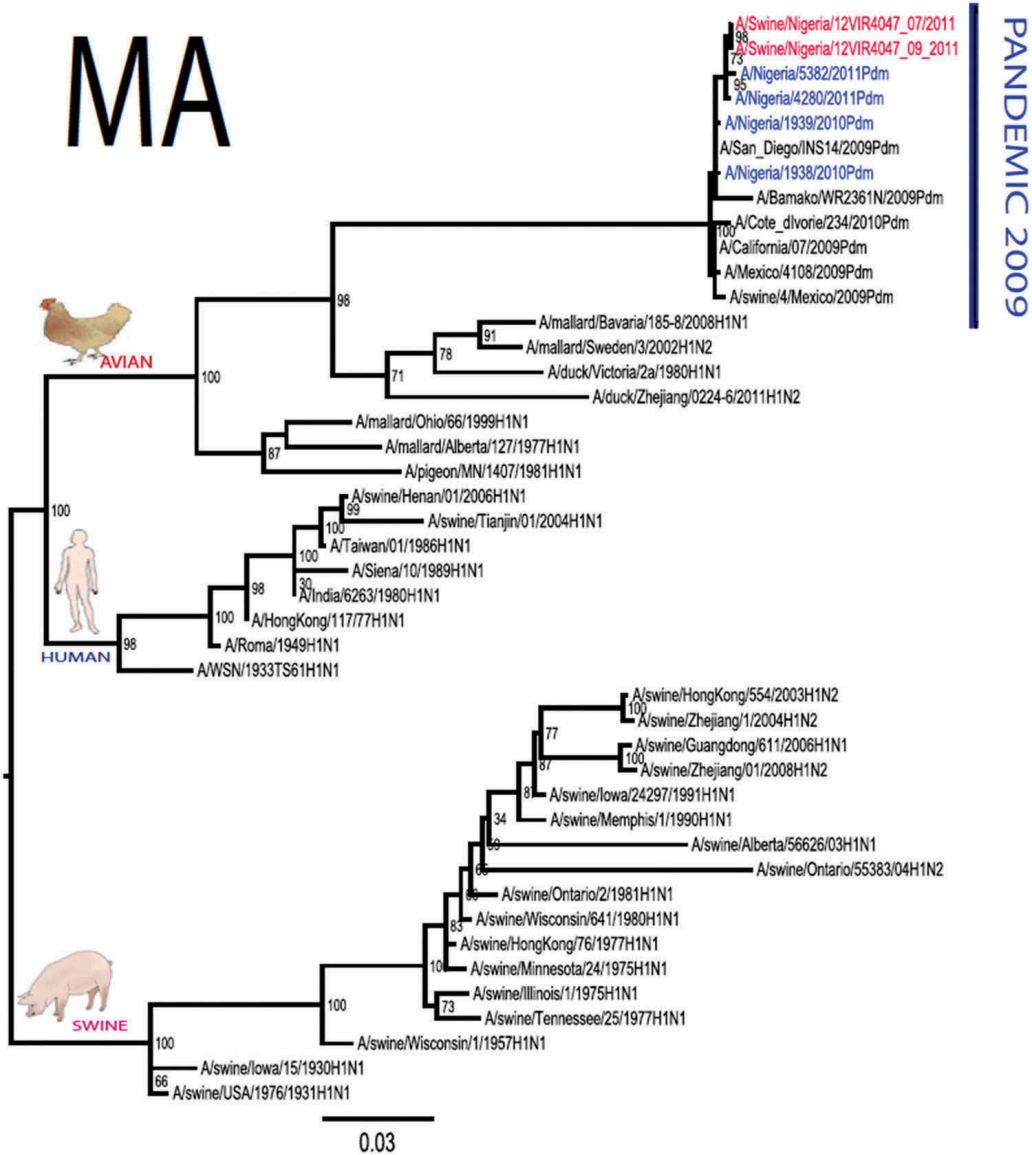
Continued.

**Figure 1.**
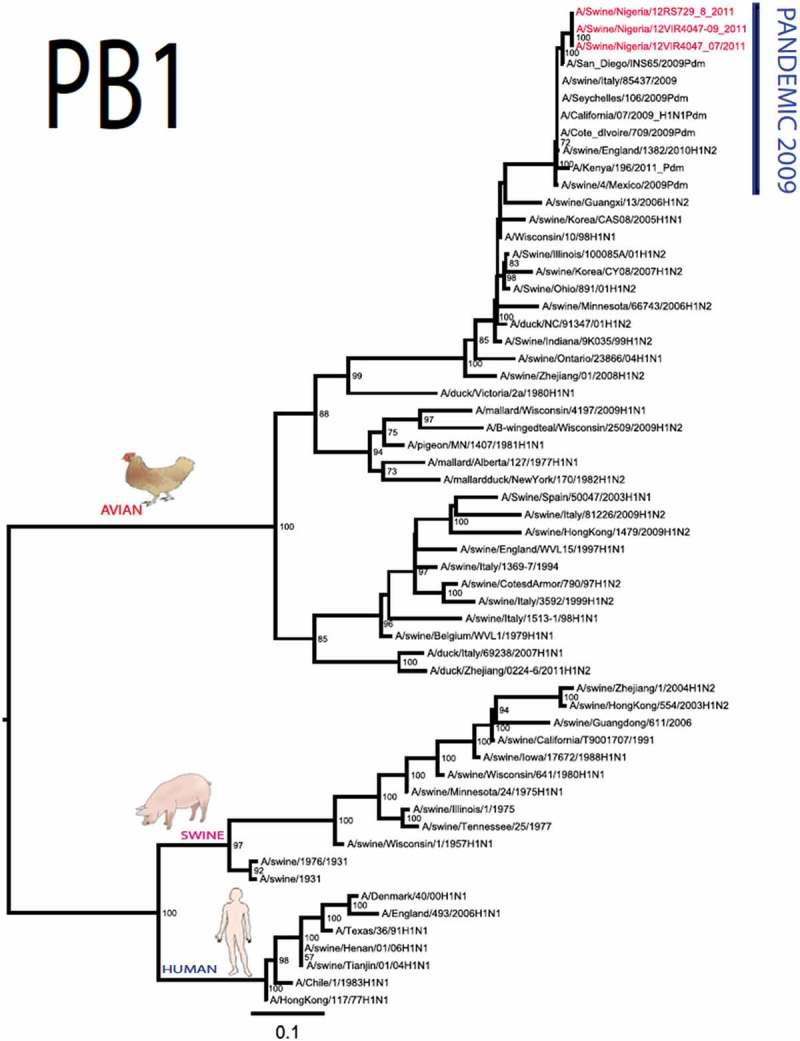
Continued.

**Figure 1.**
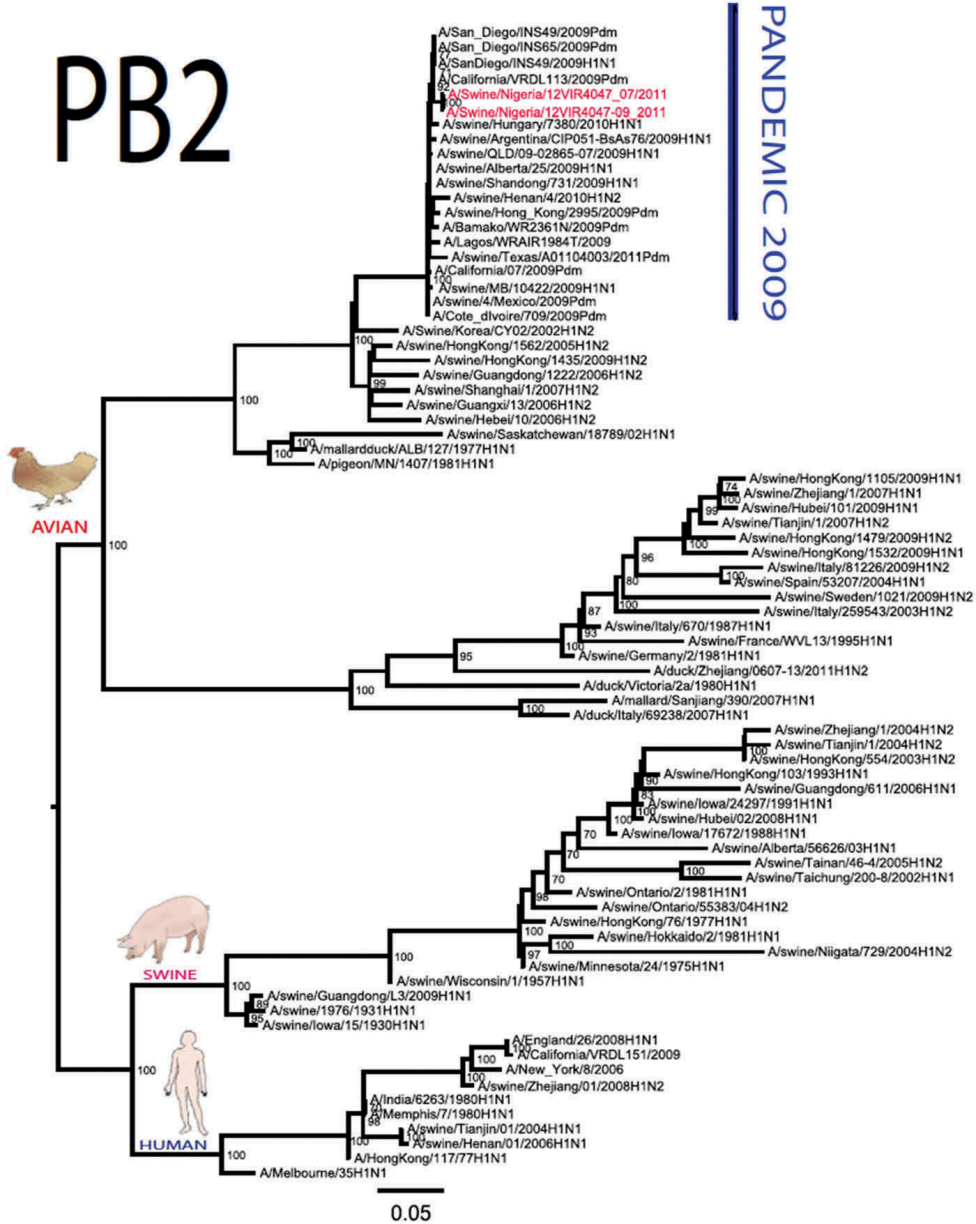
Continued.

**Figure 1.**
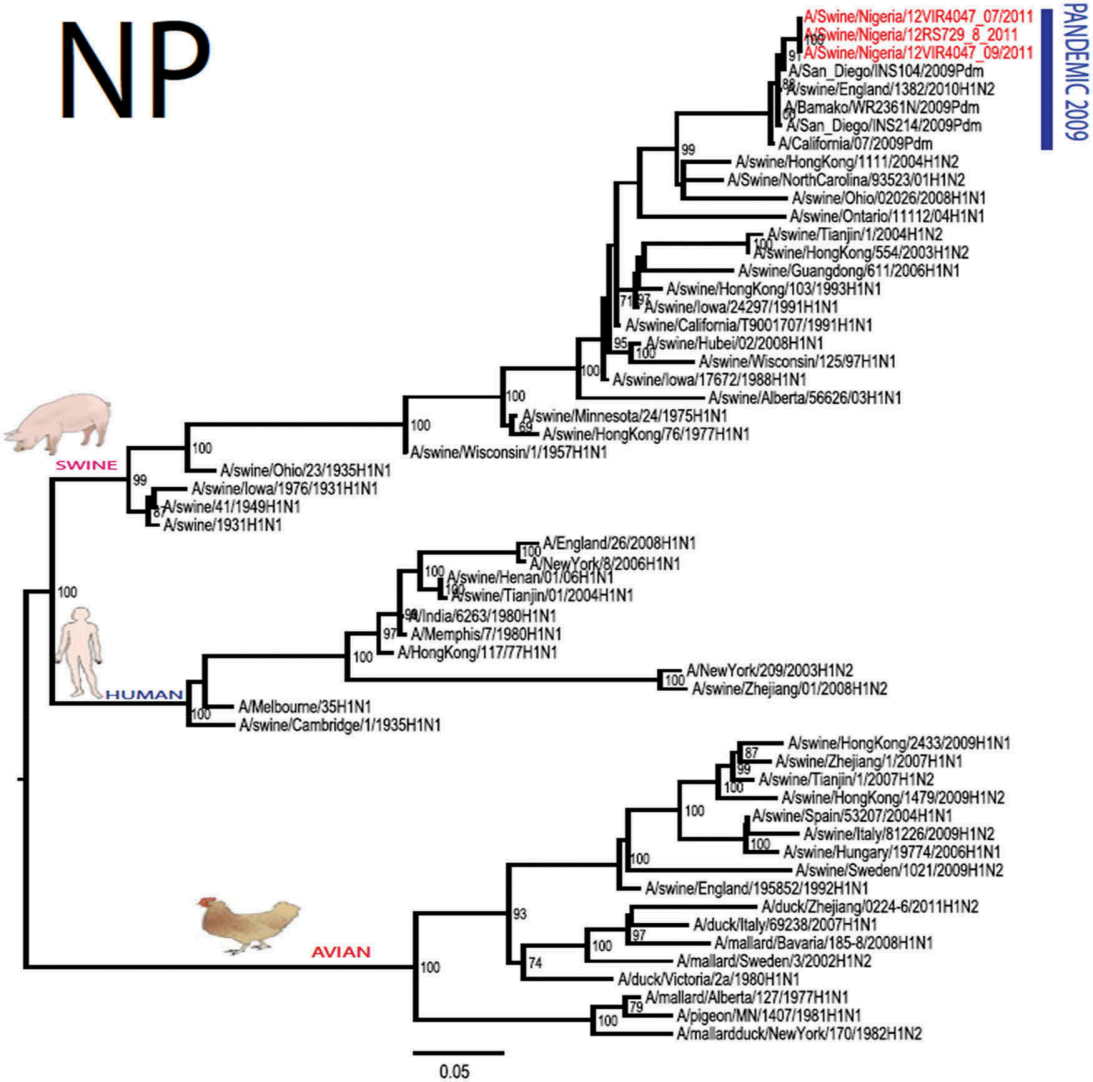
Continued.

**Figure 1.**
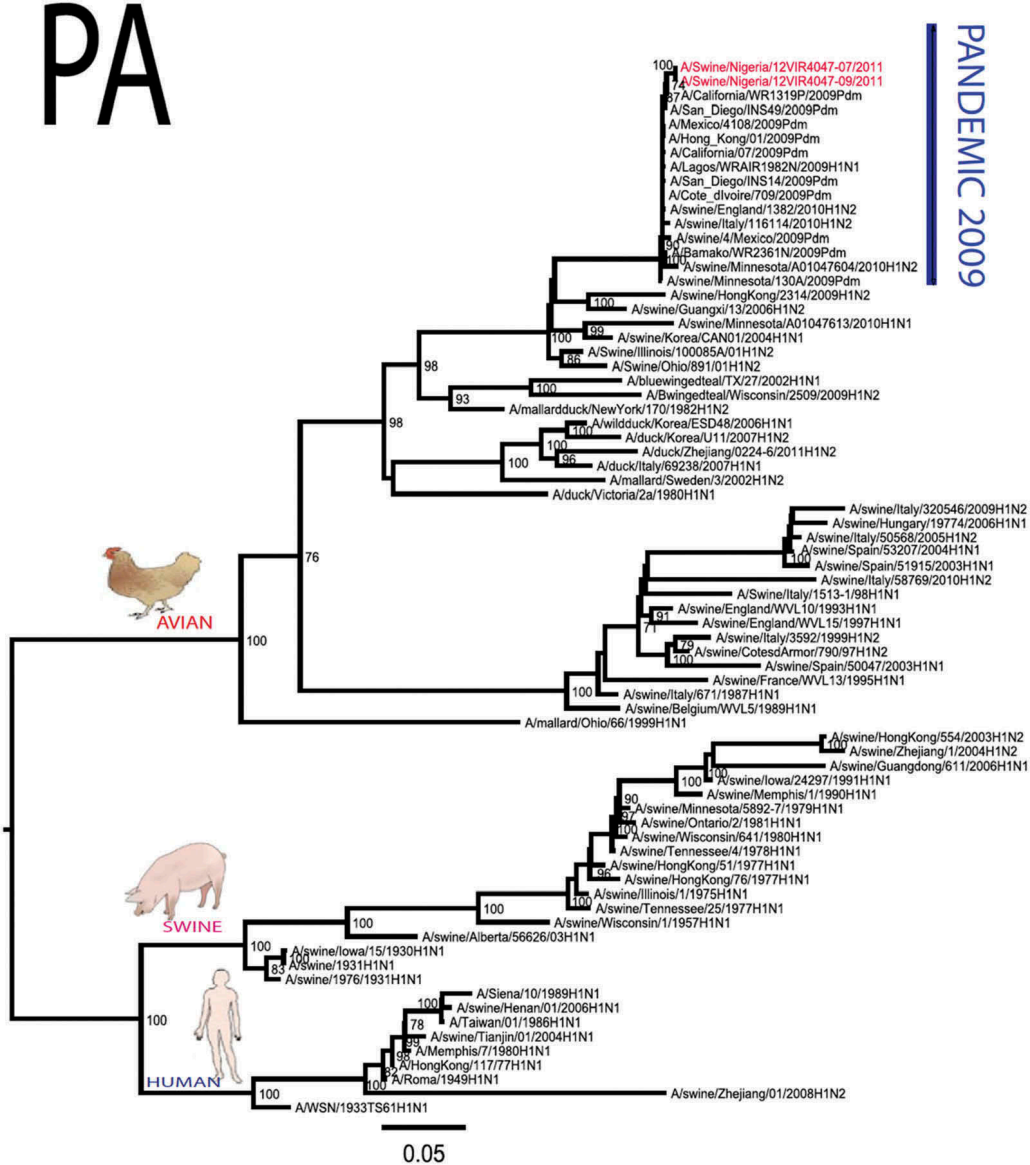
Continued.

>

**Figure 1.**
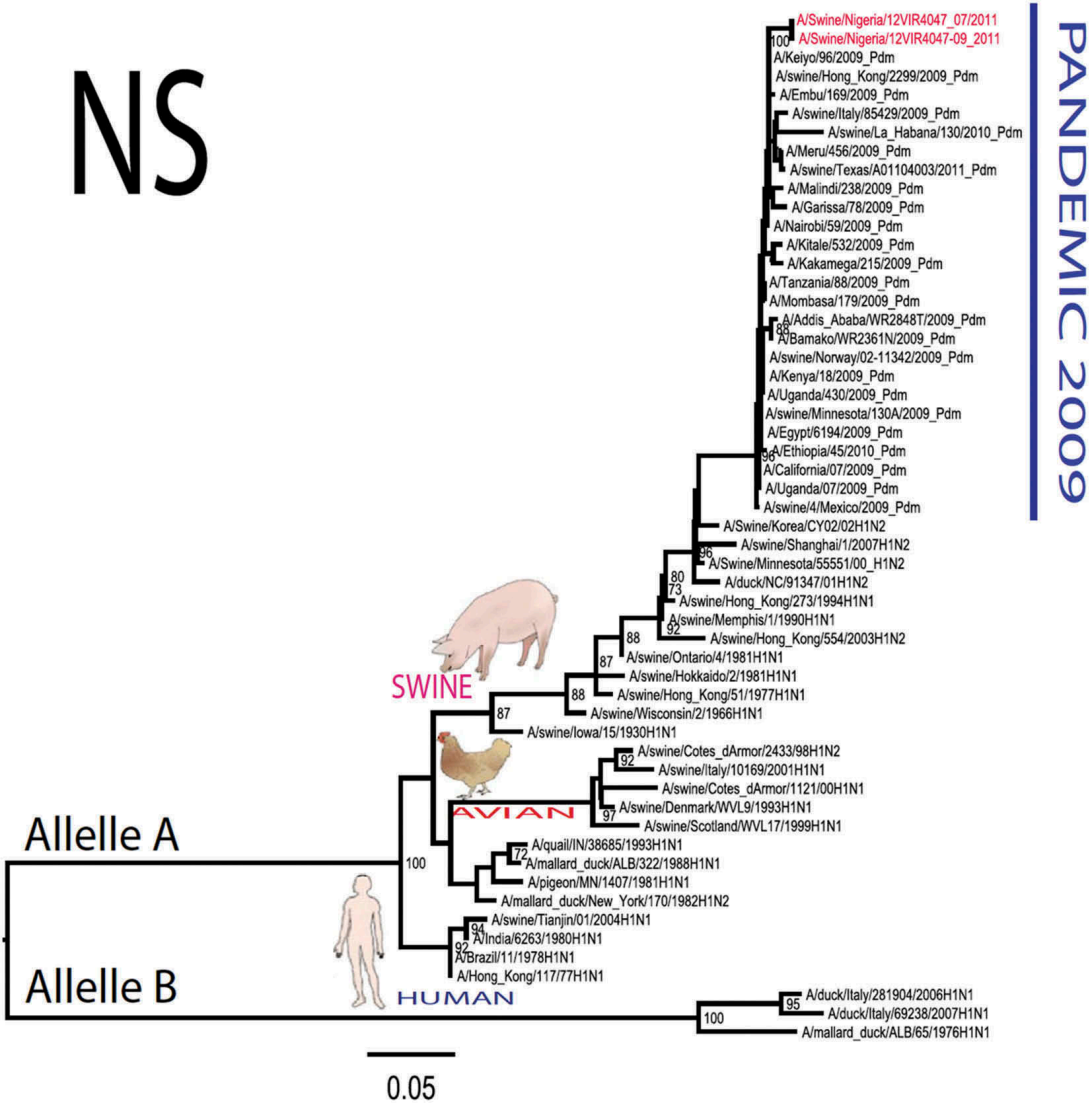
Continued.

All three isolates were sequenced and used to construct the phylogenetic trees and all of them clustered similarly with other viruses in the databases but only one was deposited in GenBank to reduce redundancy.

To gain more information about the phylogenetic relatedness of the sequenced viruses in this study, sequences from GISAID platform were retrieved and compared. The HA gene of A/swine/Nigeria/12VIR4047/2011 and the other Nigeria swine isolates are closely related to A/Nigeria/4280/2011, A/Ghana/601/2011 and A/Cameroon/EID/07/11/1870/2011 deposited in GISAID, all of which are human isolates of influenza H1N1pdm09 virus clustering together in the phylogenetic tree (Figure 1). However, nucleotide alignment of the HA gene of the swine viruses from Nigeria differed by thirteen nucleotides and three amino acid substitutions at position N138S, Q240R (H1 numbering) and I312V compared to earlier H1N1pdm09 isolates from humans in Nigeria, Cameroon, Ghana and the prototype A/California/07/2009(H1N1) (Table 1).

**Table 1.** Amino acid substitutions in A/swine/Nigeria/12VIR4047/2011 genes relative to A/California/07/2009 H1N1.

A/swine/Nigeria12VIR4047/2011	Amino acid substitutions relative to A/California/07/2009H1N1
HA	P100S, S138N, Q240R, I312Y, I338Y
NA	V106I, N248D, Y351F, S439G
MA	No substitution
PB1	M111I, K736G
PB2	D527G
PA	V14I, P224S, I505V, K716Q
NP	T23S, V100I, L122Q, D375N, T396A
NS	G28V, W102C, Q109K, I111M, I123V, W187E, G189D, V235M, S246N

Sequence analysis of the NA gene relative to A/California/07/2009 also revealed amino acid substitutions at positions V106I, N248D, Y351F, S439G. S439G substitution in the amino acid is also unique to A/Swine/Nigeria/VIR44-09/2009. All other reference viruses has S in this position. The PB2 protein of Nigerian isolate of H1N1pdm09 had D527G amino acid substitution, for PB1 G replaced K in position 736, for NS gene amino acid substitutions between A/Swine/Nigeria and prototype A/California include G28V, W103C, Q109K, I111M, I123V, W187E, G189D, V234M, S246N. The PA gene also had four substitutions presented in Table 1. Most of these mutations are silent without a yet to be described significance. There were no observed substitutions in the matrix gene except S31N that has been described for most influenza A/H1N1pdm09 isolates as the predominant amantadine-resistant mutation in M2.

## Discussion

The full genome of three H1N1pdm09 isolates from pigs in Nigeria that were analysed in this study were most similar to influenza H1N1pdm09 sequences from human hosts as obtained in GenBank BLAST. This is also supported phylogenetically with the close similarities of A/swine/Nigeria/12VIR4047/2011(H1N1) with A/Nigeria/4280/2011(H1N1), A/Ghana/601/2011(H1N1) and A/Cameroon/EID/07/11/1870/2011(H1N1) which are viral sequences from human. Ghana and Cameroon are neigbouring West African countries with social and economic linkages as well as human/material traffic with Nigeria, hence the potential for transboundary transmission of infectious pathogens. Interestingly, earlier detection of H1N1pdm09 influenza virus in pig was reported in Cameroon [11], but the swine virus is less related to the isolates from pigs in Nigeria unlike the human isolates in the same country (Figure 1), this observation suggests different introduction of the pandemic virus into the pig populations in the region (Cameroon, Nigeria and other countries including isolates from Kenya and Togo that were later detected from pigs in Africa). The human isolate of H1N1pdm09 influenza virus (A/Nigeria/4280/2011(H1N1)) detected from nasal swab sample by molecular methods at the CDC Influenza Laboratory in Abuja was sequenced and deposited in GISAID by CDC Atlanta (https://platform.gisaid.org/epi3/frontend#312740). That sample was traced to Kano in Northern Nigeria, a major commercial center with direct human and material traffic with Lagos in the southern part of the country where this study was carried out. Similar cases and detection of H1N1pdm09 in human were also reported in Nigeria about the same period (2009 to 2011) suggesting prior circulation of the virus in humans before probable transmission of the virus to pigs [12]. This virus or their progenitors may have circulated either subclinically in human population to have enabled interspecies transmission to pigs that is strongly favoured by prevailing ecological factors and poor biosecurity in the study site where pigs of all ages are mixed together and pig handlers do not have sufficient knowledge and use of personal protective equipment (PPE) [9]. However, this assertion is difficult to prove because there were no sufficient surveillance data on swine influenza or H1N1pdm09 in the country prior to 2009 global outbreaks. Available but limited surveillance data also makes it difficult to trace back the circulation of these viruses in humans and animals within the country.

The observed transmission and circulation of influenza H1N1pdm09 from humans to pigs resulted in the emergence of a virus with few amino acid substitutions in the animal host. These substitutions include the 240 position (H1 numbering) of H1N1pdm09 HA which is occupied by either amino acid glutamine (Q) in 95% of cases including in H1, H2 and H3 or arginine (R) in only about 5% of cases (Matrosovich *et al*., 2000). Most H1N1pdm09 influenza virus circulating globally including those detected in human host in Nigeria have Q in position 240 (Q226 -H3 numbering) [13]. This is subsituted by arginine (R) in the swine isolates from Nigeria. This mutation may also have been selected as a result of replication in embryonated egg as suggested by [14] where human viruses in chicken-embryonated eggs resulted in selection of variants with amino acid substitutions near the HA receptor-binding sites.

Though the observation of host-induced selection of variants was reported for Q226R or D225G of earlier human H1N1pdm09 viruses, this study observed Q240R substitution. Previous studies found four important positions (204, 239, 240 and 242) to be involved in the specific binding capacity of HA to the host cell receptor [15]. Thus the glutamine to arginine substitution at the receptor-binding site of HA could also have been positively selected for preferential binding to alpha 2,3-linked sialic acid also found in swine respiratory tract. Whether the Q240R substitution is a natural substitution or avian host induced can be further investigated by culture in mammalian cells or animal experimental studies. However, if the Nigerian swine isolates have preference for alpha 2,3 receptors also found in pigs in addition to alpha 2,6, given the presence and interaction of avian species with pigs in farms investigated, the risk of transmission of H1N1pdm09 to other susceptible host and the emergence of novel and reassortant strains is high and need to be monitored. In a recent investigation of slaughtered pigs at abattoir in Nigeria, avian H5N1 was confirmed to have been transmitted to pigs that are also subclinically exposed to H1N1pdm [16]. Taken together, these results show the risks of zoonotic transmission in multiple directions between human and animals that is favoured by intensive husbandry practices and poor biosecurity.

This first and till date only report of complete genome sequencing of H1N1pdm09 virus in Nigeria represent the first full data in the GenBank from Africa and provides baseline genomic data on swine influenza virus circulating in pigs in Nigeria and probably the rest of West African agro-ecological zone as later reported in Kenya, Togo, Uganda and further detection in Nigeria and Ghana [17–19]. This likely reverse zoonoses also revealed the epidemiological intricacies in the circulation and transmission of influenza virus at the human-animal interface whereas infectious pathogens can readily cross interspecies barrier and are transmitted from human to animals with potentials for re-transmission from animals to human. It is, however, important to note that Nigerian health-care workers and the general public have limited knowledge of the risk of transmission of influenza virus from animals to human and from human to animals [20]

## Conclusion

Given the genetic characteristics of influenza viruses, the continued circulation of H1N1pdm09 viruses in pigs in Nigeria and other parts of West Africa provide opportunities for antigenic drift and shift and co-circulating with human, avian or swine influenza viruses. This increases the risk of emergence of influenza virus variants with enhanced intra and interspecies transmission and pathogenicity in a region recognized as a zoonotic hotspot. This thus warrants continued ecological and molecular surveillance.
