# Supplementary material for: Complete genome sequencing of H1N1pdm09 swine influenza isolates from Nigeria reveals likely reverse zoonotic transmission at the human-animal interface in intensive piggery
Source: Infect Ecol Epidemiol. 2019 Dec 2;9(1):1696632. doi: 10.1080/20008686.2019.1696632 (PMC6896411; doi:10.1080/20008686.2019.1696632)
Supplement: Supplemental Material [file ZIEE_A_1696632_SM5061.zip › Supplementary/Supplementary file.docx]

**Supplementary File:**

**Primers sequence of bases used in the full genome sequencing of A/swine/Nigeria/12VIR4047-09/2011(H1N1)**

**Influenza A/H1N1 gene Segment (PB2) Primers sequence bases**

Fragment 1

1- 538-

F: 5‘- tgt aaa acg gcc agt agc aaa agc agg tca att- ̳3

R: 5‘- cag gaa aca gct atg acc gct ttg rct aay atc rtc att- ̳3

Fragment 2

328- 816-

F: 5‘- tgt aaa acg acg gcc agt gtr aca tgg tgg aay aga a- ̳3

F: 5‘-cag gaa aca gct atg acc cca aar ctg aag gay gar ctg at - ̳3

Fragment 3

487- 1019

F: 5‘- tgt aaa acg gcc agt cct ggt cay gca gat ctc ag- ̳3

F: 5‘- cag gaa aca gct atg acc cct cta act gct ttt ayc cat - ̳3

Fragment 4

713- 1289

F: 5‘- tgt aaa acg acg gcc agt ccr acw gaa gaa gct gt- ̳3

F: 5 – cag gaa aca gct atg acc cct cta act gct ttt ayc atg caa- ̳3

Fragment 5

946- 1509-

F: 5‘- tgt aaa acg acg gtt agt ccr acw gaa gaa caa gct gt- ̳3 F: 5‘- cag gaa aca gct atg acc gga gta ttc atc yat att cat- ̳3

Fragment 6

1169- 1740-

F: 5‘- tgt aaa acg acg gtt agt aag caa cca gta gat tgt ttc a- ̳3 F: 5‘- cag gaa aca gct atg acc ctg aga cca ytg aat ttt rat a- ̳3

Fragment 7

1447- 2186-

F:5‘-tgt aaa acg acg gcc agt cca agy acc gag atg tca atg aga- ̳3 F:5‘-cag gaa aca gct atg acc ttr ctc art tca ttg atg ct- ̳3

Fragment 8

1683- 2341-

F:5‘-tgt aaa acg acg gcc agt caa tac cta yca rtg gat cat cag aa- ̳3 F:5‘-cag gaa aca gct atg acc tag aaa caa ggt cgt t- ̳3

**Influenza A/H1N1 gene segment (PB1)Primers sequence bases**

Fragment 1

22- 477-

F: 5‘- tgt aaa acg acg gcc agt agt aaa agt agg tca att- ̳3 R: 5‘- cag gaa aca gct atg acc ctr aaw act atr gtg tt- ̳3

Fragment 2

233- 843-

F: 5‘- cag gaa aca gct atg acc gtt caa gct ttt crc awa tg- ̳3 F: 5‘- cag gaa aca gct atg acc gaa akg gga grc tgg tgt tta - ̳3

Fragment 3

389- 1041-

F: 5‘-tgt aaa acg acg gtt agt aca agr gtg gac aaa tra c - ̳3

F: 5‘-cag gaa aca gct atg acc ctg aac cay tra ggy tga ttt - ̳3

Fragment 4

711- 1278-

F: 5‘- tgt aaa acg gcc agt tga aca cca tga cca acg a- ̳3

F: 5 – cag gaa aca gct atg acc ttg aac atg ccc atc aty cca gg- ̳3

Fragment 5

974- 1566-

F: 5‘-tgt aaa acy acg gcc agt aat caa aay cct mga atg tt - ̳3 F: 5‘- cag gaa aca gct atg acc agc tcc atg ctr aaa ttr gc- ̳3

Fragment 6

1139- 1659-

F: 5‘-tgt aaa acg acg gcc agt caa ata ccy gca gar atg ctagc - ̳3 F: 5‘-cag gaa aca gct atg acc cca agr tca ctg ttt atc at – ̳3

Fragment 7

1489- 1954

F: 5‘-tgt aaa acg acg gcc agt atg agy aaa aag aag tcy ta - ̳3 F: 5‘- cag gaa aca gct atg acc tca aty tcy tta tgg gtg ac– ̳3

Fragment 8

1532 2321

F: 5‘-tgt aaa acg acg gcc agt gcy aat tty agc atg gag ct - ̳3 F: 5‘- cag gaa aca gct atg acc agt aga aac aag gca ttt– ̳3

**Influenza A/H1N1 gene segment (PA) Primers sequence bases**

Fragment 1

0- 493-

F: 5‘-tgt aaa acg acg gcc agt agc aaa agc agg tac tga t - ̳3

R: 5‘-cag gaa aca gct atg acc tar tck gcc ttt gtg gcc att tt - ̳3

Fragment 2

235- 756-

F: 5‘-tgt aaa acg acg gcc agt cca aat gca ctk tta aar cac aga tt R- ̳3 F: 5‘-cag gaa aca gct atg acc tga gaa agc ctg ccc tca atg - ̳3

Fragment 3

361- 989-

F: 5‘-tgt aaa acg acg gcc agt tat gay tac aar gag aa - ̳3

F: 5‘- cag gaa aca gct atg acc ggt tct ttc cat cca aag aat gtt- ̳3

Fragment 4

702- 1292-

F: 5‘- tgt aaa acg acg gcc agt tgc mtt gar aat ttt agr acc ta- ̳3 F: 5 –cag gaa aca gct atg acc tcr cak gcc ttg tgg aac tca tt - ̳3

Fragment 5

894- 1662-

F: 5‘- tgt aaa acg acg gcc agt aaa ttr agc aat gar gay cca- ̳3 F: 5‘-cag gaa ata gct atg acc tcm agt cty ggg tca gtg ag - ̳3

Fragment 6

1204- 2037-

F: 5‘-tgt aaa acg acg gcc agt taa gcg att tra agt aat atg a - ̳3 F: 5‘- cag gaa aca gct atg acc aay ccy tcy aat tgt ggm gat c– ̳3

Fragment 7

1444- 2057

F: 5‘-tgt aaa acg acg gcc agt aat gca tcc tgt gca gca atg ga - ̳3 F: 5‘-cag gaa aca gct atg acc ttg tcc cta aga gcc tga aca a– ̳3

Fragment 8

1787 2233

F: 5‘- tgt aaa acg acg gcc agt atg aar tgg gga atg gag atg ag - ̳3 F: 5‘-cag gaa aca gct atg acc agt aga aac aag gta cct ttt– ̳3

**Influenza A/H1N1 gene segment (HA) Primers sequence bases**

Fragment 1

1- 461-

F: 5‘- tgt aaa acg atg gcc agt ata cga cta gta aaa gca ggg g - ̳3 R: 5‘- cag gaa aca gct atg acc tca tga ttg ggt cay ga- ̳3

Fragment 2

351- 943-

F: 5‘- tgt aaa acg acg gtt agt acr tgt tac ccw ggr gat ttc a - ̳3 F: 5‘- cag gaa aca gct atg acc gaa akg gga grc tgg tgt tta - ̳3

Fragment 3

379- 1204-

F: 5‘- tgt aaa acg acg gcc agt atg acg acc tra gag agc a - ̳3 F: 5‘- cag gaa aca gct atg acc caa tgg crt tyt gtg tgc tc - ̳3

Fragment 4

736- 1340-

F: 5‘- tgt aaa acg acg gcc agt agt atg rac tat tac tgg ac - ̳3 F: 5 - cag gaa aca gct atg acc ttc tkc att rta wgt cca aa - ̳3

Fragment 5

1124- 1541-

F: 5‘- tgt aaa acg acg gtt agt tgg atg gta ygg tta yca yca g- ̳3 F: 5‘- cag gaa aca gct atg acc tca taa gty cca ttt ytg a - ̳3

Fragment 6

1204- 1778-

F: 5‘- tgt aaa acg acg gcc agt aag atg aay acr car ttc aca g- ̳3 F: 5‘-cag gaa aca gct atg act gtg tca gta gaa aca agg gtg ttt - ̳3

**Influenza A/H1N1 gene segment (NP ) Primers sequence bases**

Fragment 1

1- 553-

F: 5‘-tgt aaa acg acg gcc agt cag ggt aga taa tca ctc ac - ̳3 R: 5‘-cag gaa aca gct atg acc aga gca cat yct ggg atc cat - ̳3

Fragment 2

296- 757-

F: 5‘- tgt aaa acg acg gcc agt atg gtr ctc tct gct ttt gat ga - ̳3

F: 5‘- cag gaa aca gct atg acc ttt gtc cag ctg ttt gaa att tyc ctt t- ̳3

Fragment 3

513- 1042

F: 5‘-tgt aaa acg acg gcc agt tgg tat tch att ttr aat gat - ̳3 F: 5‘-cag gaa aca gct atg acg ctg rct ctt gtg tgc dgg - ̳3

Fragment 4

619- 1177

F: 5‘- tgt aaa acg acg gcc agt gct gca gtc aar gga rt- ̳3

F: 5 –cag gaa aca gct atg acc aag cra ttt gta cyc ctc tag t- ̳3

Fragment 5

925- 1565-

F: 5‘- tgt aaa acg acg gcc agt cct gcy tgt gyg tam gga c- ̳3 F: 5‘- cag gaa aca gct atg acc agg aga aac aag ggt att ttt c- ̳3

**Influenza A/H1N1 gene segment (NA) Primers sequence bases**

Fragment 1

1- 600-

F: 5‘-tgt aaa acg acg gcc agt agc aaa agc agg agt - ̳3 R: 5‘- cag gaa aca gct atg acc ctg gat crg aaa ttc c- ̳3

Fragment 2

318- 740-

F: 5‘- tgt aaa acg acg gcc agt tac aca aaa gac aay agc - ̳3 F: 5‘- cag gaa aca gct atg aac ggr cca tcg gtc att atg - ̳3

Fragment 3

536- 1063-

F: 5‘-tgt aaa acg acg gcc agt ggt cag caa gcg cat gyc atg a - ̳3 F: 5‘-cag gaa aca gct atg acc cat aty tgt atg aaa acc - ̳3

Fragment 4

726- 1346-

F: 5‘- tgt aaa acg acg gcc agt aat ggr car gcc tcr tac aa- ̳3 F: 5 –cag gaa aca gct atg acc gct gct ycc rct atg caa gat- ̳3

Fragment 5

941- 1452-

F: 5‘- tgt aaa acg acg gcc agt tag gat aca tca gca gtg g- ̳3 F: 5‘- cag gaa aca gct atg acc agt aga aac aag gag- ̳3

**Influenza A/H1N1 gene segment (M) Primers sequence bases**

Fragment 1

0- 473-

F: 5‘- tgt aaa acg acg gcc agt agc aaa agc agg tag - ̳3 R: 5‘- cag gaa aca gct atg acc gca atc tgy tca cak gt- ̳3

Fragment 2

223- 750-

F: 5‘- tgt aaa acg acg gcc agt cac cgt gcc cag tga gcg - ̳3 F: 5‘- cag gaa aca gct atg acc tca ttt gaa ycg ctg cat- ̳3

Fragment 3

383- 1027-

F: 5‘-tgt aaa acg acg gcc agt tcg gct ggm gca ctt gcc agt tg - ̳3 F: 5‘-cag gaa aca gct atg acc agt agm aac aag gta gt - ̳3

**Influenza A/H1N1 gene segment (NS) Primers sequence bases**

Fragment 1

24- 482-

F: 5‘-tgt aaa acg acg gcc agt agc aaa agc agg gtg aca aag aca - ̳3 R: 5‘-cag gaa aca gct atg acc tcg gtg aaa gcc ctt a - ̳3

Fragment 2

250- 890-

F: 5‘- tgt aaa acg acg gcc agt tga ggc ayt taa aat gat ca- ̳3

F: 5‘- cag gaa aca gct atg acc agt aga aac aag ggt gtt ttt tat- ̳3

Fragment 3

418- 742-

F: 5‘- tgt aaa acg acg gcc agt aaa gcc aay ttc agt tgg - ̳3 F: 5‘- cag gaa aca gct atg acc ttc aat aag cca tct ta- ̳3
