# Supplementary material for: Complete genome sequencing of H1N1pdm09 swine influenza isolates from Nigeria reveals likely reverse zoonotic transmission at the human-animal interface in intensive piggery
Source: Infect Ecol Epidemiol. 2019 Dec 2;9(1):1696632. doi: 10.1080/20008686.2019.1696632 (PMC6896411; doi:10.1080/20008686.2019.1696632)
Supplement: Supplemental Material [file ZIEE_A_1696632_SM5061.zip › Supplementary/Supplementary_file_2_for prod.docx]

**Data availability Statement:**

Genomic data generated in this study has been deposited in the Genbank under accession number : **JX482555.1 to JX482560.1 and JX442481.1 & JX442481**

The data below is publicly available in the link:

<https://www.ncbi.nlm.nih.gov/genomes/FLU/Database/nph-select.cgi#mainform>

**Supplementary file 2: Gene sequences and record in the Genbank for A/H1N1pdm09 isolated from Nigerian pig**

**PB1- GenBank: JX482555.1**

Influenza A virus (A/swine/Nigeria/12VIR4047-09/2011(H1N1)) segment 1 polymerase PB2 (PB2) gene, complete cds

GenBank: JX482555.1

FASTA Graphics

Go to:

LOCUS JX482555 2280 bp cRNA linear VRL 04-SEP-2012

DEFINITION Influenza A virus (A/swine/Nigeria/12VIR4047-09/2011(H1N1)) segment

1 polymerase PB2 (PB2) gene, complete cds.

ACCESSION JX482555

VERSION JX482555.1

DBLINK BioProject: PRJNA37813

KEYWORDS .

SOURCE Influenza A virus (A/swine/Nigeria/12VIR4047-09/2011(H1N1))

ORGANISM Influenza A virus (A/swine/Nigeria/12VIR4047-09/2011(H1N1))

Viruses; ssRNA viruses; ssRNA negative-strand viruses;

Orthomyxoviridae; Influenzavirus A.

REFERENCE 1 (bases 1 to 2280)

AUTHORS Meseko,C.A., Heidari,A., Monne,I., Fusaro,A., Cattoli,G.,

Odaibo,G.N., Bakarey,A.S., Olaleye,D.O. and Capua,I.

TITLE Molecular characterization of pandemic A/H1N1 2009 influenza virus

isolated in pigs in Lagos, Nigeria

JOURNAL Unpublished

REFERENCE 2 (bases 1 to 2280)

AUTHORS Meseko,C.A., Heidari,A., Monne,I., Fusaro,A., Cattoli,G.,

Odaibo,G.N., Bakarey,A.S., Olaleye,D.O. and Capua,I.

TITLE Direct Submission

JOURNAL Submitted (13-AUG-2012) FAO/OIE Reference Laboratory for Newcastle

Disease and Avian Influenza, OIE Collaborating Center for Diseases

at the Human-Animal Interface, Istituto Zooprofilattico

Sperimentale delle Venezie, Viale dell'Universita 10, Legnaro,

Padova 35020, Italy

COMMENT ##Assembly-Data-START##

Sequencing Technology :: Sanger dideoxy sequencing

##Assembly-Data-END##

FEATURES Location/Qualifiers

source 1..2280

/organism="Influenza A virus

(A/swine/Nigeria/12VIR4047-09/2011(H1N1))"

/mol_type="viral cRNA"

/strain="A/swine/Nigeria/12VIR4047-09/2011"

/serotype="H1N1"

/isolation_source="nasal swab"

/host="swine"

/db_xref="taxon:1217303"

/segment="1"

/country="Nigeria: Lagos"

/collection_date="27-May-2011"

/note="lineage: swl; passage details: E1"

gene 1..2280

/gene="PB2"

CDS 1..2280

/gene="PB2"

/codon_start=1

/product="polymerase PB2"

/protein_id="AFQ90306.1"

/translation="MERIKELRDLMSQSRTREILTKTTVDHMAIIKKYTSGRQEKNPA

LRMKWMMAMRYPITADKRIMDMIPERNEQGQTLWSKTNDAGSDRVMVSPLAVTWWNRN

GPTTSTVHYPKVYKTYFEKVERLKHGTFGPVHFRNQVKIRRRVDTNPGHADLSAKEAQ

DVIMEVVFPNEVGARILTSESQLAITKEKKEELQDCKIAPLMVAYMLERELVRKTRFL

PVAGGTGSVYIEVLHLTQGTCWEQMYTPGGEVRNDDVDQSLIIAARNIVRRAAVSADP

LASLLEMCHSTQIGGVRMVDILRQNPTEEQAVDICKAAIGLRISSSFSFGGFTFKRTS

GSSVKKEEEVLTGNLQTLKIRVHEGYEEFTMVGRRATAILRKATRRLIQLIVSGRDEQ

SIAEAIIVAMVFSQEDCMIKAVRGDLNFVNRANQRLNPMHQLLRHFQKDAKVLFQNWG

IESIDNVMGMIGILPDMTPSTEMSLRGIRVSKMGVDEYSSTERVVVSIDRFLRVRDQR

GNVLLSPEEVSETQGTEKLTITYSSSMMWEINGPESVLVNTYQWIIRNWEIVKIQWSQ

GPTMLYNKMEFEPFQSLVPKATRSRYSGFVRTLFQQMRDVLGTFDTVQIIKLLPFAAA

PPEQSRMQFSSLTVNVRGSGLRILVRGNSPVFNYNKATKRLTVLGKDAGALTEDPDEG

TSGVESAVLRGFLILGKEDKRYGPALSINELSNLAKGEKANVLIGQGDVVLVMKRKRD

SSILTDSQTATKRIRMAIN"

ORIGIN

1 atggagagaa taaaagaact gagagatcta atgtcgcagt cccgcactcg cgagatactc

61 actaagacca ctgtggacca tatggccata atcaaaaagt acacatcagg aaggcaagag

121 aagaaccccg cactcagaat gaagtggatg atggcaatga gatacccaat tacagcagac

181 aagagaataa tggacatgat tccagaaagg aatgaacaag gacaaaccct ctggagcaaa

241 acaaacgacg ctggatcaga ccgagtgatg gtatcacctc tggccgtaac atggtggaat

301 aggaatggcc caacaacaag tacagttcat taccctaagg tatataaaac ttatttcgaa

361 aaggtcgaaa ggttgaaaca tggcaccttc ggccctgtcc acttcagaaa tcaagttaaa

421 ataaggagga gagttgatac aaaccctggc catgcagatc tcagtgccaa ggaggcacaa

481 gatgtgatta tggaagttgt tttcccaaat gaagtggggg caagaatact gacatcagag

541 tcacagctgg caataacaaa agagaagaaa gaagagctcc aggattgtaa aattgctccc

601 ttgatggtgg cgtacatgct agaaagagaa ttggtccgca aaacaaggtt tctcccagta

661 gccggcggaa caggcagtgt ttatattgaa gtgttgcact taacccaagg gacgtgctgg

721 gagcagatgt acactccagg aggagaagtg agaaatgatg atgttgacca aagtttgatt

781 atcgctgcta gaaacatagt aagaagagca gcagtgtcag cagacccatt agcatctctc

841 ttggaaatgt gccacagcac acagattgga ggagtaagga tggtagacat ccttagacag

901 aatccaactg aggaacaagc cgtagacata tgcaaggcag caatagggtt gagaattagc

961 tcatctttca gttttggtgg gttcactttc aaaaggacaa gcggatcatc agtcaagaaa

1021 gaagaagaag tgctaacagg caaccttcaa acactgaaaa taagagtaca tgaagggtat

1081 gaagaattca caatggttgg gagaagagca acagctattc tcagaaaggc aaccaggaga

1141 ttgatccagt tgatagtaag cgggagagac gagcagtcaa ttgctgaggc aataattgtg

1201 gccatggtat tctcacaaga ggattgcatg atcaaggcag ttaggggcga tctgaacttt

1261 gtcaataggg caaaccagcg actgaacccc atgcaccaac tcttgaggca tttccaaaaa

1321 gatgcaaaag tgcttttcca gaactgggga attgaatcca tcgacaatgt gatgggaatg

1381 atcggaatac tgcccgacat gactccaagc acggagatgt cgctgagagg gataagagtc

1441 agcaaaatgg gagtagatga atactccagc acggagagag tggtggtgag tattgaccga

1501 tttttaaggg ttagagatca aagagggaac gtactattgt ctcccgaaga agtcagtgaa

1561 acgcaaggaa ctgagaagtt gacaataact tattcgtcat caatgatgtg ggagatcaat

1621 ggccctgagt cagtgctagt caacacttat caatggataa tcaggaactg ggaaattgtg

1681 aaaattcaat ggtcacaagg tcccacaatg ttatacaaca aaatggaatt tgaaccattt

1741 cagtctcttg tccctaaggc aaccagaagc cggtacagtg gattcgtaag gacactgttc

1801 cagcaaatga gggatgtgct tgggacattt gacactgtcc aaataataaa acttctcccc

1861 tttgctgctg ctccaccaga acagagtagg atgcaatttt cctcattgac tgtgaatgtg

1921 agaggatcag ggttgaggat actggtaaga ggcaattctc cagtattcaa ttacaacaag

1981 gcaaccaaac gacttacagt tcttggaaag gatgcaggtg cattgactga agatccagat

2041 gaaggcacat ctggggtgga gtctgctgtc ctgagaggat ttctcatttt gggcaaagaa

2101 gacaagagat atggcccagc attaagcatc aatgaactga gcaatcttgc aaaaggagag

2161 aaagctaatg tgctaattgg gcaaggggac gtagtgttgg taatgaaacg aaaacgggac

2221 tctagcatac ttactgacag ccagacagcg accaaaagaa ttcggatggc catcaattag

**PB1- GenBank: JX482556.1**

Influenza A virus (A/swine/Nigeria/12VIR4047-09/2011(H1N1)) segment 2 polymerase PB1 (PB1) gene, complete cds; and nonfunctional PB1-F2 protein (PB1-F2) gene, complete sequence

GenBank: JX482556.1

FASTA Graphics

LOCUS JX482556 2274 bp cRNA linear VRL 04-SEP-2012

DEFINITION Influenza A virus (A/swine/Nigeria/12VIR4047-09/2011(H1N1)) segment

2 polymerase PB1 (PB1) gene, complete cds; and nonfunctional PB1-F2

protein (PB1-F2) gene, complete sequence.

ACCESSION JX482556

VERSION JX482556.1

DBLINK BioProject: PRJNA37813

KEYWORDS .

SOURCE Influenza A virus (A/swine/Nigeria/12VIR4047-09/2011(H1N1))

ORGANISM Influenza A virus (A/swine/Nigeria/12VIR4047-09/2011(H1N1))

Viruses; ssRNA viruses; ssRNA negative-strand viruses;

Orthomyxoviridae; Influenzavirus A.

REFERENCE 1 (bases 1 to 2274)

AUTHORS Meseko,C.A., Heidari,A., Monne,I., Fusaro,A., Cattoli,G.,

Odaibo,G.N., Bakarey,A.S., Olaleye,D.O. and Capua,I.

TITLE Molecular characterization of pandemic A/H1N1 2009 influenza virus

isolated in pigs in Lagos, Nigeria

JOURNAL Unpublished

REFERENCE 2 (bases 1 to 2274)

AUTHORS Meseko,C.A., Heidari,A., Monne,I., Fusaro,A., Cattoli,G.,

Odaibo,G.N., Bakarey,A.S., Olaleye,D.O. and Capua,I.

TITLE Direct Submission

JOURNAL Submitted (13-AUG-2012) FAO/OIE Reference Laboratory for Newcastle

Disease and Avian Influenza, OIE Collaborating Center for Diseases

at the Human-Animal Interface, Istituto Zooprofilattico

Sperimentale delle Venezie, Viale dell'Universita 10, Legnaro,

Padova 35020, Italy

COMMENT ##Assembly-Data-START##

Sequencing Technology :: Sanger dideoxy sequencing

##Assembly-Data-END##

FEATURES Location/Qualifiers

source 1..2274

/organism="Influenza A virus

(A/swine/Nigeria/12VIR4047-09/2011(H1N1))"

/mol_type="viral cRNA"

/strain="A/swine/Nigeria/12VIR4047-09/2011"

/serotype="H1N1"

/isolation_source="nasal swab"

/host="swine"

/db_xref="taxon:1217303"

/segment="2"

/country="Nigeria: Lagos"

/collection_date="27-May-2011"

/note="lineage: swl; passage details: E1"

gene 1..2274

/gene="PB1"

CDS 1..2274

/gene="PB1"

/codon_start=1

/product="polymerase PB1"

/protein_id="AFQ90307.1"

/translation="MDVNPTLLFLKIPAQNAISTTFPYTGDPPYSHGTGTGYTMDTVN

RTHQYSEKGKWTTNTETGAPQLNPIDGPLPEDNEPSGYAQTDCVLEAMAFLEESHPGI

FENSCLETIEVVQQTRVDKLTQGRQTYDWTLNRNQPAATALANTIEVFRSNGLTANES

GRLIDFLKDVMESMNKEEIEITTHFQRKRRVRDNMTKKMVTQRTIGKKKQRLNKRGYL

IRALTLNTMTKDAERGKLKRRAIATPGMQIRGFVYFVETLARSICEKLEQSGLPVGGN

EKKAKLANVVRKMMTNSQDTEISFTITGDNTKWNENQNPRMFLAMITYITRNQPEWFR

NILSMAPIMFSNKMARLGKGYMFESKRMKIRTQIPAEMLASIDLKYFNESTKKKIEKI

RPLLIDGTASLSPGMMMGMFNMLSTVLGVSILNLGQKKYTKTIYWWDGLQSSDDFALI

VNAPNHEGIQAGVDRFYRTCKLVGINMSKKKSYINKTGTFEFTSFFYRYGFVANFSME

LPSFGVSGVNESADMSIGVTVIKNNMINNDLGPATAQMALQLFIKDYRYTYRCHRGDT

QIQTRRSFELKKLWDQTQSKVGLLVSDGGPNLYNIRNLHIPEVCLKWELMDDDYRGRL

CNPLNPFVSHKEIDSVNNAVVMPAHGPAKSMEYDAVATTHSWIPKRNRSILNTSQRGI

LEDEQMYQKCCNLFEKFFPSSSYRRPVGISSMVEAMVSRARIDARVDFESGRIGKEEF

SEIMKICSTIEELRRQK"

gene 95..367

/gene="PB1-F2"

misc_feature 95..367

/gene="PB1-F2"

/note="nonfunctional PB1-F2 protein due to mutation"

ORIGIN

1 atggatgtca atccgactct acttttccta aaaattccag cgcaaaatgc cataagcacc

61 acattccctt atactggaga tcctccatac agccatggaa caggaacagg atacaccatg

121 gacacagtaa acagaacaca ccaatactca gaaaagggaa agtggacgac aaacacagag

181 actggtgcac cccagctcaa cccgattgat ggaccactac ctgaggataa tgaaccaagt

241 gggtatgcac aaacagactg tgttctagag gccatggctt tccttgaaga atcccaccca

301 ggaatatttg agaattcatg ccttgaaaca attgaagttg ttcaacaaac aagggtagat

361 aaactaactc aaggtcgcca gacttatgat tggacattaa acagaaatca accggcagct

421 actgcattgg ccaacaccat agaagtcttt agatcgaatg gcctaacagc taatgagtca

481 ggaaggctaa tagatttctt aaaggatgta atggaatcaa tgaacaaaga ggaaatagag

541 ataactaccc actttcaaag aaaaaggaga gtaagagaca acatgaccaa gaagatggtc

601 acgcaaagaa caataggaaa gaaaaaacaa agactgaata aaagaggcta tctaataaga

661 gcactgacat taaatacgat gaccaaagat gcagagagag gcaagttaaa aagaagggct

721 atcgcaacac ctgggatgca gattagaggt ttcgtatact ttgttgaaac tttagctagg

781 agcatttgcg aaaagcttga acagtctggg ctcccagtag ggggcaatga aaagaaagcc

841 aaactggcaa atgttgtgag aaagatgatg actaattcac aagacacaga gatttctttc

901 acaatcactg gggacaacac taagtggaat gaaaatcaaa atcctcgaat gttcctggcg

961 atgattacat atatcaccag aaatcaaccc gagtggttca gaaacatcct gagcatggca

1021 cctataatgt tctcaaacaa aatggcaaga ctagggaaag ggtacatgtt cgagagtaaa

1081 agaatgaaga ttcgaacaca aataccagca gaaatgctag caagcattga cctgaagtac

1141 ttcaatgaat caacaaagaa aaaaattgag aaaataaggc ctcttctaat agatggcaca

1201 gcatcactga gtcctgggat gatgatgggc atgttcaaca tgctcagtac ggtcttggga

1261 gtctcgatac tgaatcttgg acaaaagaaa tacaccaaga caatatactg gtgggatggg

1321 ctccaatcat ccgacgattt tgctctcata gtgaatgcac caaaccatga gggaatacaa

1381 gcaggagtgg acagattcta caggacctgc aagttagtgg gaatcaacat gagcaaaaag

1441 aagtcctata taaataagac agggacattt gaattcacaa gcttttttta tcgctatgga

1501 tttgtggcta attttagcat ggagctaccc agctttggag tgtctggagt aaatgaatca

1561 gctgacatga gtattggagt aacagtgata aaaaacaaca tgataaacaa tgaccttgga

1621 cctgcaacgg cccagatggc tcttcaactg ttcatcaaag actacagata cacatatagg

1681 tgccataggg gagacacaca aattcaaacg aggagatcat ttgagttaaa gaagctgtgg

1741 gatcaaaccc aatcaaaggt agggctatta gtatcagatg gaggaccaaa cttatacaat

1801 ataaggaatc ttcacattcc tgaagtctgc ttaaaatggg agctaatgga tgatgattat

1861 cggggaagac tttgtaatcc cctgaatccc tttgtcagtc ataaagagat tgattctgta

1921 aacaatgctg tggtgatgcc agcccatggt ccagccaaaa gcatggaata tgatgccgtt

1981 gcaactacac attcctggat tcccaagagg aatcgttcta ttctcaacac aagccaaagg

2041 ggaattcttg aggatgaaca gatgtaccag aagtgctgca atctattcga gaaattcttc

2101 cctagcagtt catataggag accggttgga atttctagca tggtggaggc catggtgtct

2161 agggcccgga ttgatgccag ggtcgacttt gagtctggac ggatcgggaa ggaagagttc

2221 tctgagatca tgaagatctg ttccaccatt gaagaactca gacggcaaaa ataa

//

**PA- GenBank: JX482557.1**

Influenza A virus (A/swine/Nigeria/12VIR4047-09/2011(H1N1)) segment 3 polymerase PA (PA) gene, complete cds

GenBank: JX482557.1

FASTA Graphics

LOCUS JX482557 2151 bp cRNA linear VRL 04-SEP-2012

DEFINITION Influenza A virus (A/swine/Nigeria/12VIR4047-09/2011(H1N1)) segment

3 polymerase PA (PA) gene, complete cds.

ACCESSION JX482557

VERSION JX482557.1

DBLINK BioProject: PRJNA37813

KEYWORDS .

SOURCE Influenza A virus (A/swine/Nigeria/12VIR4047-09/2011(H1N1))

ORGANISM Influenza A virus (A/swine/Nigeria/12VIR4047-09/2011(H1N1))

Viruses; ssRNA viruses; ssRNA negative-strand viruses;

Orthomyxoviridae; Influenzavirus A.

REFERENCE 1 (bases 1 to 2151)

AUTHORS Meseko,C.A., Heidari,A., Monne,I., Fusaro,A., Cattoli,G.,

Odaibo,G.N., Bakarey,A.S., Olaleye,D.O. and Capua,I.

TITLE Molecular characterization of pandemic A/H1N1 2009 influenza virus

isolated in pigs in Lagos, Nigeria

JOURNAL Unpublished

REFERENCE 2 (bases 1 to 2151)

AUTHORS Meseko,C.A., Heidari,A., Monne,I., Fusaro,A., Cattoli,G.,

Odaibo,G.N., Bakarey,A.S., Olaleye,D.O. and Capua,I.

TITLE Direct Submission

JOURNAL Submitted (13-AUG-2012) FAO/OIE Reference Laboratory for Newcastle

Disease and Avian Influenza, OIE Collaborating Center for Diseases

at the Human-Animal Interface, Istituto Zooprofilattico

Sperimentale delle Venezie, Viale dell'Universita 10, Legnaro,

Padova 35020, Italy

COMMENT ##Assembly-Data-START##

Sequencing Technology :: Sanger dideoxy sequencing

##Assembly-Data-END##

FEATURES Location/Qualifiers

source 1..2151

/organism="Influenza A virus

(A/swine/Nigeria/12VIR4047-09/2011(H1N1))"

/mol_type="viral cRNA"

/strain="A/swine/Nigeria/12VIR4047-09/2011"

/serotype="H1N1"

/isolation_source="nasal swab"

/host="swine"

/db_xref="taxon:1217303"

/segment="3"

/country="Nigeria: Lagos"

/collection_date="27-May-2011"

/note="lineage: swl; passage details: E1"

gene 1..2151

/gene="PA"

CDS 1..2151

/gene="PA"

/codon_start=1

/product="polymerase PA"

/protein_id="AFQ90308.1"

/translation="MEDFVRQCFNPMIIELAEKAMKEYGEDPKIETNKFAAICTHLEV

CFMYSDFHFIDERGESIIVESGDPNALLKHRFEIIEGRDRIMAWTVVNSICNTTGVEK

PKFLPDLYDYKENRFIEIGVTRREVHIYYLEKANKIKSEKTHIHIFSFTGEEMATKAD

YTLDEESRARIKTRLFTIRQEMASRSLWDSFRQSERGEETIEEKFEITGTMRKLADQS

LPPNFSSLENFRAYVDGFEPNGCIEGKLSQMSKEVNAKIEPFLRTTPRPLRLPDGPLC

HQRSKFLLMDALKLSIEDPSHEGEGIPLYDAIKCMKTFFGWKEPNIVKPHEKGINPNY

LMAWKQVLAELQDIENEEKIPRTKNMKRTSQLKWALGENMAPEKVDFDDCKDVGDLKQ

YDSDEPEPRSLASWVQNEFNKACELTDSSWIELDEIGEDVAPIEHIASMRRNYFTAEV

SHCRATEYIMKGVYINTALLNASCAAMDDFQLIPMISKCRTKEGRRKTNLYGFIVKGR

SHLRNDTDVVNFVSMEFSLTDPRLEPHKWEKYCVLEIGDMLLRTAIGQVSRPMFLYVR

TNGTSKIKMKWGMEMRRCLLQSLQQIESVIEAESSVKEKDMTKEFFENKSETWPIGES

PRGVEEGSIGKVCRTLLAKSVFNSLYASPQLEGFSAESRKLLLIVQALRDNLEPGTFD

LGGLYEAIEECLINDPWVLLNASWFNSFLTHALQ"

ORIGIN

1 atggaagact ttgtgcgaca atgcttcaat ccaatgatca tcgagcttgc ggaaaaggca

61 atgaaagaat atggggaaga tccgaaaatc gaaactaaca agtttgctgc aatatgcaca

121 catttggaag tttgtttcat gtattcggat ttccatttca tcgacgaacg gggtgaatca

181 ataattgtag aatctggtga cccgaatgca ctattgaagc accgatttga gataattgaa

241 ggaagagacc gaatcatggc ctggacagtg gtgaacagta tatgtaacac aacaggggta

301 gagaagccta aatttcttcc tgatttgtat gattacaaag agaaccggtt cattgaaatt

361 ggagtaacac ggagggaagt ccacatatat tacctagaga aagccaacaa aataaaatct

421 gagaagacac acattcacat cttttcattc actggagagg agatggccac caaagcggac

481 tacacccttg acgaagagag cagggcaaga atcaaaacta ggcttttcac tataagacaa

541 gaaatggcca gtaggagtct atgggattcc tttcgtcagt ccgaaagagg cgaagagaca

601 attgaagaaa aatttgagat tacagggact atgcgcaagc ttgccgacca aagtctccca

661 ccgaacttct ccagccttga aaactttaga gcctatgtag atggattcga gccgaacggc

721 tgcattgagg gcaagctttc ccaaatgtca aaagaagtga acgccaaaat tgagccattc

781 ttgaggacga caccacgccc cctcagattg cctgatgggc ctctttgcca tcagcggtca

841 aagttcctgc tgatggatgc tctgaaatta agtattgaag acccgagtca cgagggggag

901 ggaataccac tatatgatgc aatcaaatgc atgaagacat tctttggctg gaaagagccc

961 aacatagtca aaccacatga gaaaggcata aatcccaatt acctcatggc ttggaagcag

1021 gtgctagcag agctacagga cattgaaaat gaagagaaga tcccaaggac aaagaacatg

1081 aagagaacaa gccaattgaa gtgggcactc ggtgaaaata tggcaccaga aaaagtagac

1141 tttgatgact gcaaagatgt tggagacctt aaacagtatg acagtgatga gccagagccc

1201 agatctctag caagctgggt ccaaaatgaa ttcaataagg catgtgaatt gactgattca

1261 agctggatag aacttgatga aataggagaa gatgttgccc cgattgaaca tatcgcaagt

1321 atgaggagga actattttac agcagaagtg tctcactgca gggctactga atacataatg

1381 aagggagtgt acataaatac ggccttgctc aatgcatcct gtgcagccat ggatgacttt

1441 cagctgatcc caatgataag caaatgtagg accaaagaag gaagacggaa aacaaacctg

1501 tatgggttca ttgtaaaagg aaggtctcat ttgagaaatg atactgatgt ggtgaacttt

1561 gtaagtatgg agttctcact cactgacccg agactggagc cacacaaatg ggaaaaatac

1621 tgtgttcttg aaataggaga catgctcttg aggactgcga taggccaagt gtcgaggccc

1681 atgttcctat atgtgagaac caatggaacc tccaagatca agatgaaatg gggcatggaa

1741 atgaggcgct gccttcttca gtctcttcag cagattgaga gcgtgattga ggccgagtct

1801 tctgtcaaag agaaagacat gaccaaggaa ttctttgaaa acaaatcgga aacatggcca

1861 atcggagagt cacccagggg agtggaggaa ggctctattg ggaaagtgtg caggacctta

1921 ctggcaaaat ctgtattcaa cagtctatat gcgtctccac aacttgaggg gttttcggct

1981 gaatcgagaa aattgcttct cattgttcag gcacttaggg acaacctgga acctggaacc

2041 ttcgatcttg gggggctata tgaagcaatc gaggagtgcc tgattaatga tccctgggtt

2101 ttgcttaatg catcttggtt caactccttc ctcacacatg cactgcagta g

//

**HA- GenBank: JX442481.1**

Influenza A virus (A/swine/Nigeria/12VIR4047-09/2011(H1N1)) segment 4 hemagglutinin (HA) gene, complete cds

GenBank: JX442481.1

FASTA Graphics

Go to:

LOCUS JX442481 1701 bp cRNA linear VRL 29-AUG-2012

DEFINITION Influenza A virus (A/swine/Nigeria/12VIR4047-09/2011(H1N1)) segment

4 hemagglutinin (HA) gene, complete cds.

ACCESSION JX442481

VERSION JX442481.1

KEYWORDS .

SOURCE Influenza A virus (A/swine/Nigeria/12VIR4047-09/2011(H1N1))

ORGANISM Influenza A virus (A/swine/Nigeria/12VIR4047-09/2011(H1N1))

Viruses; ssRNA viruses; ssRNA negative-strand viruses;

Orthomyxoviridae; Influenzavirus A.

REFERENCE 1 (bases 1 to 1701)

AUTHORS Meseko,C.A., Heidari,A., Monne,I., Fusaro,A., Cattoli,G.,

Odaibo,G.N., Bakarey,A.S., Olaleye,D.O. and Capua,I.

TITLE Molecular characterization of pandemic A/H1N1 2009 influenza virus

isolated in pigs in Lagos, Nigeria

JOURNAL Unpublished

REFERENCE 2 (bases 1 to 1701)

AUTHORS Meseko,C.A., Heidari,A., Monne,I., Fusaro,A., Cattoli,G.,

Odaibo,G.N., Bakarey,A.S., Olaleye,D.O. and Capua,I.

TITLE Direct Submission

JOURNAL Submitted (31-JUL-2012) OIE/FAO Reference Laboratory for Newcastle

Disease and Avian Influenza, OIE Collaborating Center for Diseases

at the Human-Animal Interface, Istituto Zooprofilattico

Sperimentale delle Venezie, Viale dell'Universita 10, Legnaro,

Padova 35020, Italy

COMMENT ##Assembly-Data-START##

Sequencing Technology :: Sanger dideoxy sequencing

##Assembly-Data-END##

FEATURES Location/Qualifiers

source 1..1701

/organism="Influenza A virus

(A/swine/Nigeria/12VIR4047-09/2011(H1N1))"

/mol_type="viral cRNA"

/strain="A/swine/Nigeria/12VIR4047-09/2011"

/serotype="H1N1"

/host="swine"

/db_xref="taxon:1217303"

/segment="4"

/country="Nigeria: Lagos"

/collection_date="27-May-2011"

gene 1..1701

/gene="HA"

CDS 1..1701

/gene="HA"

/codon_start=1

/product="hemagglutinin"

/protein_id="AFQ37281.1"

/translation="MKAILVVLLYTFATTNADTLCIGYHANNSTDTVDTVLEKNVTVT

HSVNLLEDKHNGKLCKLRGVAPLHLGKCNIAGWILGNPECESLSTASSWSYIVETSSS

DNGTCYPGDFIDYEELREQLSSVSSFERFEIFPKTSSWPNHDSNKGVTAACPHAGAKS

FYKNLIWLVKKGNSYPKLSKSYINDKGKEVLVLWGIHHPSTSTDQQSLYQNADAYVFV

GTSRYSKKFKPEIAIRPKVRDREGRMNYYWTLVEPGDKITFEATGNLVVPRYAFAMER

NAGSGIIISDTPAHDCNTTCQTPKGAINTSLPFQNVHPITIGKCPKYVKSTKLRLATG

LRNVPSIQSRGLFGAIAGFIEGGWTGMVDGWYGYHHQNEQGSGYAADLKSTQNAIDKI

TNKVNSVIEKMNTQFTAVGKEFNHLEKRIENLNKKVDDGFLDIWTYNAELLVLLENER

TLDYHDSNVKNLYEKVRSQLKNNAKEIGNGCFEFYHKCDDKCMESVKNGTYDYPKYSE

EAKLNREEIDGVKLESTRIYQILAIYSTAASSLVLVVSLGAISFWMCSNGSLQCRICI

"

ORIGIN

1 atgaaggcaa tactagtagt tctgctatat acatttgcaa ccacaaatgc agacacatta

61 tgtataggtt atcatgcgaa caattcaaca gacactgtag acacagtact agaaaagaat

121 gtaacagtaa cacactctgt taaccttcta gaagacaagc ataacgggaa actatgcaaa

181 ctaagaggag tagccccatt gcatttgggt aaatgtaaca ttgctggctg gatcctggga

241 aatccagagt gtgaatcact ctccacagca agctcatggt cctacattgt ggaaacatct

301 agttcagaca atggaacgtg ttacccagga gatttcatcg attatgagga gctaagagag

361 caattgagct cagtgtcatc atttgaaagg tttgagatat tccccaagac aagttcatgg

421 cccaatcatg actcgaacaa aggtgtaacg gcagcatgtc ctcatgctgg agcaaaaagc

481 ttctacaaaa atttaatatg gctagttaaa aaaggaaatt catacccaaa gctcagcaaa

541 tcttatatta atgataaagg gaaagaagtc ctcgtgctat ggggcattca ccatccatct

601 actagtactg accaacaaag tctctatcag aatgcagatg catatgtttt tgtggggaca

661 tcaagataca gcaagaagtt caagccggaa atagcaataa gacccaaagt gagggatcga

721 gaagggagaa tgaactatta ctggacacta gtagaaccgg gagacaaaat aacattcgaa

781 gcaactggaa atctagtggt accgagatat gcattcgcga tggaaagaaa tgctggatct

841 ggtattatca tttcagatac accagcccac gattgcaata caacttgtca gacacccaaa

901 ggtgctataa acaccagcct cccatttcag aatgtacatc cgatcacaat tggaaaatgt

961 ccaaaatatg taaaaagcac aaaattgaga ctggccacag gattgaggaa tgtcccgtct

1021 attcaatcta gaggcctatt tggggccatt gccggtttca ttgaaggggg gtggacaggg

1081 atggtagatg gatggtacgg ttatcaccat caaaatgagc aggggtcagg atatgcagcc

1141 gacctgaaga gcacacagaa tgccattgac aagattacta acaaagtaaa ttctgttatt

1201 gaaaagatga atacacagtt cacagcagta ggtaaagagt tcaaccacct ggaaaaaaga

1261 atagagaatt taaataaaaa agttgatgat ggtttcctgg acatttggac ttacaatgcc

1321 gaactgttgg ttctattgga aaatgaaaga actttggact accacgattc aaatgtgaag

1381 aacttatatg aaaaggtaag aagccagtta aaaaacaatg ccaaggaaat tggaaacggc

1441 tgctttgaat tttaccacaa atgcgatgac aagtgcatgg aaagtgtcaa aaatgggact

1501 tatgactacc caaaatactc agaggaagca aaattaaaca gagaagaaat agatggggta

1561 aagctggaat caacaaggat ttaccagatt ttggcgatct attcaactgc cgccagttca

1621 ttggtactgg tagtctccct gggggcaatc agtttctgga tgtgctctaa tgggtctcta

1681 cagtgtagaa tatgtattta a

//

**NP- GenBank: JX482558.1**

Influenza A virus (A/swine/Nigeria/12VIR4047-09/2011(H1N1)) segment 5 nucleocapsid protein (NP) gene, complete cds

GenBank: JX482558.1

FASTA Graphics

LOCUS JX482558 1497 bp cRNA linear VRL 04-SEP-2012

DEFINITION Influenza A virus (A/swine/Nigeria/12VIR4047-09/2011(H1N1)) segment

5 nucleocapsid protein (NP) gene, complete cds.

ACCESSION JX482558

VERSION JX482558.1

DBLINK BioProject: PRJNA37813

KEYWORDS .

SOURCE Influenza A virus (A/swine/Nigeria/12VIR4047-09/2011(H1N1))

ORGANISM Influenza A virus (A/swine/Nigeria/12VIR4047-09/2011(H1N1))

Viruses; ssRNA viruses; ssRNA negative-strand viruses;

Orthomyxoviridae; Influenzavirus A.

REFERENCE 1 (bases 1 to 1497)

AUTHORS Meseko,C.A., Heidari,A., Monne,I., Fusaro,A., Cattoli,G.,

Odaibo,G.N., Bakarey,A.S., Olaleye,D.O. and Capua,I.

TITLE Molecular characterization of pandemic A/H1N1 2009 influenza virus

isolated in pigs in Lagos, Nigeria

JOURNAL Unpublished

REFERENCE 2 (bases 1 to 1497)

AUTHORS Meseko,C.A., Heidari,A., Monne,I., Fusaro,A., Cattoli,G.,

Odaibo,G.N., Bakarey,A.S., Olaleye,D.O. and Capua,I.

TITLE Direct Submission

JOURNAL Submitted (13-AUG-2012) FAO/OIE Reference Laboratory for Newcastle

Disease and Avian Influenza, OIE Collaborating Center for Diseases

at the Human-Animal Interface, Istituto Zooprofilattico

Sperimentale delle Venezie, Viale dell'Universita 10, Legnaro,

Padova 35020, Italy

COMMENT ##Assembly-Data-START##

Sequencing Technology :: Sanger dideoxy sequencing

##Assembly-Data-END##

FEATURES Location/Qualifiers

source 1..1497

/organism="Influenza A virus

(A/swine/Nigeria/12VIR4047-09/2011(H1N1))"

/mol_type="viral cRNA"

/strain="A/swine/Nigeria/12VIR4047-09/2011"

/serotype="H1N1"

/isolation_source="nasal swab"

/host="swine"

/db_xref="taxon:1217303"

/segment="5"

/country="Nigeria: Lagos"

/collection_date="27-May-2011"

/note="lineage: swl; passage details: E1"

gene 1..1497

/gene="NP"

CDS 1..1497

/gene="NP"

/codon_start=1

/product="nucleocapsid protein"

/protein_id="AFQ90309.1"

/translation="MASQGTKRSYEQMETGGERQDASEIRASVGRMIGGIGRFYIQMC

TELKLSDYDGRLIQNSITIERMVLSAFDERRNKYLEEHPSAGKDPKKTGGPIYRRIDG

KWMRELILYDKEEIRRVWRQANNGEDATAGLTHIMIWHSNLNDATYQRTRALVRTGMD

PRMCSLMQGSTLPRRSGAAGAAVKGVGTIAMELIRMIKRGINDRNFWRGENGRRTRVA

YERMCNILKGKFQTAAQRAMMDQVRESRNPGNAEIEDLIFLARSALILRGSVAHKSCL

PACVYGLAVASGHDFEREGYSLVGIDPFKLLQNSQVVSLMRPNENPAHKSQLVWMACH

SAAFEDLRVSSFIRGKKVIPRGKLSTRGVQIASNENVETMNSNTLELRSRYWAIRTRS

GGNANQQKASAGQISVQPTFSVQRNLPFERATVMAAFSGNNEGRTSDMRTEVIRMMES

AKPEDLSFQGRGVFELSDEKATNPIVPSFDMSNEGSYFFGDNAEEYDS"

ORIGIN

1 atggcgtctc aaggcaccaa acgatcatat gaacaaatgg agactggtgg ggagcgccag

61 gatgcctcag aaatcagagc atctgtcgga agaatgattg gtggaatcgg gagattctac

121 atccaaatgt gcactgaact caaactcagt gattatgatg gacgactaat ccagaatagc

181 ataacaatag agaggatggt gctttctgct tttgatgaga gaagaaataa atacctagaa

241 gagcatccca gtgctgggaa ggaccctaag aaaacaggag gacccatata tagaagaata

301 gacggaaagt ggatgagaga actcatcctt tatgacaaag aagaaataag gagagtttgg

361 cgccaagcaa ataatggcga agatgcaaca gcaggtctta ctcatatcat gatttggcat

421 tccaatctga atgatgccac atatcagaga acaagagcgc ttgttcgcac cggaatggat

481 cccagaatgt gctctctaat gcaaggttca acacttccca gaaggtctgg tgccgcaggt

541 gctgcggtga aaggagttgg aacaatagca atggagttaa tcagaatgat caaacgtgga

601 atcaatgacc gaaatttctg gaggggtgaa aatggacgaa ggacaagggt tgcttatgaa

661 agaatgtgca atatcctcaa aggaaaattt caaacagctg cccagagggc aatgatggat

721 caagtaagag aaagtcgaaa cccaggaaac gctgagattg aagacctcat tttcctggca

781 cggtcagcac tcattctgag gggatcagtt gcacataaat cctgcctgcc tgcttgtgtg

841 tatgggcttg cagtagcaag tgggcatgac tttgaaaggg aagggtactc actggtcggg

901 atagacccat tcaaattact ccaaaacagc caagtggtca gcctgatgag accaaatgaa

961 aacccagctc acaagagtca attggtgtgg atggcatgcc actctgctgc atttgaagat

1021 ttgagagtat caagtttcat aagaggaaag aaagtgattc caagaggaaa gctttccaca

1081 agaggagtcc agattgcttc aaatgagaat gtggaaacca tgaactccaa taccctagaa

1141 ctaagaagca gatactgggc cataaggacc aggagtggag gaaatgccaa tcaacaaaag

1201 gcatccgcag gccagatcag tgtgcagcct acattctcag tgcagcgaaa tctccctttt

1261 gaaagagcaa ccgttatggc agcattcagc gggaacaatg aaggacggac atccgacatg

1321 cgaacagaag ttataagaat gatggaaagt gcaaagccag aagatttgtc ctttcagggg

1381 cggggagtct tcgagctctc agacgaaaag gcaacgaacc cgatcgtgcc ttcctttgac

1441 atgagtaatg aagggtctta tttcttcgga gacaatgcag aggagtatga cagttga

//

**NA- GenBank: JX442482.1**

Influenza A virus (A/swine/Nigeria/12VIR4047-09/2011(H1N1)) segment 6 neuraminidase (NA) gene, complete cds

GenBank: JX442482.1

FASTA Graphics

LOCUS JX442482 1410 bp cRNA linear VRL 29-AUG-2012

DEFINITION Influenza A virus (A/swine/Nigeria/12VIR4047-09/2011(H1N1)) segment

6 neuraminidase (NA) gene, complete cds.

ACCESSION JX442482

VERSION JX442482.1

KEYWORDS .

SOURCE Influenza A virus (A/swine/Nigeria/12VIR4047-09/2011(H1N1))

ORGANISM Influenza A virus (A/swine/Nigeria/12VIR4047-09/2011(H1N1))

Viruses; ssRNA viruses; ssRNA negative-strand viruses;

Orthomyxoviridae; Influenzavirus A.

REFERENCE 1 (bases 1 to 1410)

AUTHORS Meseko,C.A., Heidari,A., Monne,I., Fusaro,A., Cattoli,G.,

Odaibo,G.N., Bakarey,A.S., Olaleye,D.O. and Capua,I.

TITLE Molecular characterization of pandemic A/H1N1 2009 influenza virus

isolated in pigs in Lagos, Nigeria

JOURNAL Unpublished

REFERENCE 2 (bases 1 to 1410)

AUTHORS Meseko,C.A., Heidari,A., Monne,I., Fusaro,A., Cattoli,G.,

Odaibo,G.N., Bakarey,A.S., Olaleye,D.O. and Capua,I.

TITLE Direct Submission

JOURNAL Submitted (01-AUG-2012) OIE/FAO Reference Laboratory for Newcastle

Disease and Avian Influenza, OIE Collaborating Center for Diseases

at the Human-Animal Interface, Istituto Zooprofilattico

Sperimentale delle Venezie, Viale dell'Universita 10, Legnaro,

Padova 35020, Italy

COMMENT ##Assembly-Data-START##

Sequencing Technology :: Sanger dideoxy sequencing

##Assembly-Data-END##

FEATURES Location/Qualifiers

source 1..1410

/organism="Influenza A virus

(A/swine/Nigeria/12VIR4047-09/2011(H1N1))"

/mol_type="viral cRNA"

/strain="A/swine/Nigeria/12VIR4047-09/2011"

/serotype="H1N1"

/host="swine"

/db_xref="taxon:1217303"

/segment="6"

/country="Nigeria: Lagos"

/collection_date="27-May-2011"

gene 1..1410

/gene="NA"

CDS 1..1410

/gene="NA"

/codon_start=1

/product="neuraminidase"

/protein_id="AFQ37282.1"

/translation="MNPNQKIITIGSVCMTIGMANLILQIGNIISIWISHSIQLGNQN

QIETCNQSVITYENNTWVNQTYVNISNTNFAAGQSVVSVKLAGNSSLCPVSGWAIYSK

DNSIRIGSKGDVFVIREPFISCSPLECRTFFLTQGALLNDKHSNGTIKDRSPYRTLMS

CPIGEVPSPYNSRFESVAWSASACHDGINWLTIGISGPDNGAVAVLKYNGIITDTIKS

WRNNILRTQESECACVNGSCFTVMTDGPSDGQASYKIFRIEKGKIVKSVEMNAPNYHY

EECSCYPDSSEITCVCRDNWHGSNRPWVSFNQNLEYQIGYICSGIFGDNPRPNDKTGS

CGPVSSNGANGVKGFSFKYGNGVWIGRTKSISSRNGFEMIWDPNGWTGTDNNFSIKQD

IVGINEWSGYSGSFVQHPELTGLDCIRPCFWVELIRGRPKENTIWTGGSSISFCGVNS

DTVGWSWPDGAELPFTIDK"

ORIGIN

1 atgaatccaa accaaaagat aataaccatt ggttcggtct gtatgacaat tggaatggct

61 aacctaatat tacaaattgg aaacataatc tcaatatgga ttagccactc aattcaactt

121 gggaatcaaa atcagattga aacatgcaat caaagcgtca ttacttatga aaacaacact

181 tgggtaaatc agacatatgt taacatcagc aacaccaact ttgctgctgg acagtcagtg

241 gtttccgtga aattagcggg caattcctct ctctgccctg ttagtggatg ggctatatac

301 agtaaagaca acagtataag aatcggttcc aagggggatg tgtttgtcat aagggaacca

361 ttcatatcat gctccccctt ggaatgcaga accttcttct tgacccaagg ggccttgcta

421 aatgacaaac attccaatgg aaccattaaa gacaggagcc catatcgaac cctaatgagc

481 tgtcctattg gtgaagttcc ctctccatac aactcaagat ttgagtcagt cgcttggtca

541 gcaagtgctt gtcatgatgg catcaattgg ctaacaattg gaatttctgg cccagacaat

601 ggggcagtgg ctgtgttaaa gtacaacggc ataataacag acactatcaa gagttggaga

661 aacaatatat tgagaacaca agagtccgaa tgtgcatgtg taaatggttc ttgctttact

721 gtaatgaccg atggaccaag tgatggacag gcctcataca agatcttcag aatagaaaag

781 ggaaaaatag tcaaatcagt cgaaatgaat gcccctaatt atcactatga ggaatgctct

841 tgttatcctg attctagtga aatcacatgt gtgtgcaggg ataactggca tggctcgaat

901 cgaccgtggg tgtctttcaa ccagaatctg gaatatcaga taggatacat atgcagtggg

961 attttcgggg acaatccacg ccctaatgat aagacaggca gttgtggtcc agtatcgtct

1021 aatggagcaa atggagtaaa agggttttca ttcaaatacg gcaatggtgt ttggataggg

1081 agaactaaaa gcattagttc aagaaacggt tttgagatga tttgggatcc gaacggatgg

1141 actgggacag acaataactt ctcaataaag caagatatcg taggaataaa tgaatggtca

1201 ggatatagcg ggagttttgt tcagcatcca gaactaacag ggctggattg tataagacct

1261 tgcttctggg ttgaactaat cagagggcga cccaaagaga acacaatctg gactggcggg

1321 agcagcatat ccttttgtgg tgtaaacagt gacactgtgg gttggtcttg gccagacggt

1381 gctgagttgc catttaccat tgacaagtaa

//

**MA- GenBank: JX482559.1**

Influenza A virus (A/swine/Nigeria/12VIR4047-09/2011(H1N1)) segment 7 matrix protein 2 (M2) gene, partial cds; and matrix protein 1 (M1) gene, complete cds

GenBank: JX482559.1

FASTA Graphics

LOCUS JX482559 956 bp cRNA linear VRL 04-SEP-2012

DEFINITION Influenza A virus (A/swine/Nigeria/12VIR4047-09/2011(H1N1)) segment

7 matrix protein 2 (M2) gene, partial cds; and matrix protein 1

(M1) gene, complete cds.

ACCESSION JX482559

VERSION JX482559.1

DBLINK BioProject: PRJNA37813

KEYWORDS .

SOURCE Influenza A virus (A/swine/Nigeria/12VIR4047-09/2011(H1N1))

ORGANISM Influenza A virus (A/swine/Nigeria/12VIR4047-09/2011(H1N1))

Viruses; ssRNA viruses; ssRNA negative-strand viruses;

Orthomyxoviridae; Influenzavirus A.

REFERENCE 1 (bases 1 to 956)

AUTHORS Meseko,C.A., Heidari,A., Monne,I., Fusaro,A., Cattoli,G.,

Odaibo,G.N., Bakarey,A.S., Olaleye,D.O. and Capua,I.

TITLE Molecular characterization of pandemic A/H1N1 2009 influenza virus

isolated in pigs in Lagos, Nigeria

JOURNAL Unpublished

REFERENCE 2 (bases 1 to 956)

AUTHORS Meseko,C.A., Heidari,A., Monne,I., Fusaro,A., Cattoli,G.,

Odaibo,G.N., Bakarey,A.S., Olaleye,D.O. and Capua,I.

TITLE Direct Submission

JOURNAL Submitted (13-AUG-2012) FAO/OIE Reference Laboratory for Newcastle

Disease and Avian Influenza, OIE Collaborating Center for Diseases

at the Human-Animal Interface, Istituto Zooprofilattico

Sperimentale delle Venezie, Viale dell'Universita 10, Legnaro,

Padova 35020, Italy

COMMENT ##Assembly-Data-START##

Sequencing Technology :: Sanger dideoxy sequencing

##Assembly-Data-END##

FEATURES Location/Qualifiers

source 1..956

/organism="Influenza A virus

(A/swine/Nigeria/12VIR4047-09/2011(H1N1))"

/mol_type="viral cRNA"

/strain="A/swine/Nigeria/12VIR4047-09/2011"

/serotype="H1N1"

/isolation_source="nasal swab"

/host="swine"

/db_xref="taxon:1217303"

/segment="7"

/country="Nigeria: Lagos"

/collection_date="27-May-2011"

/note="lineage: swl; passage details: E1"

gene 1..>956

/gene="M2"

CDS join(1..26,715..>956)

/gene="M2"

/codon_start=1

/product="matrix protein 2"

/protein_id="AFQ90311.1"

/translation="MSLLTEVETPTRSEWECRCSDSSDPLVIAANIIGILHLILWITD

RLFFKCIYRRFKYGLKRGPSTEGVPESMREEYQQEQHSAVDVDDG"

gene 1..759

/gene="M1"

CDS 1..759

/gene="M1"

/codon_start=1

/product="matrix protein 1"

/protein_id="AFQ90310.1"

/translation="MSLLTEVETYVLSIIPSGPLKAEIAQRLESVFAGKNTDLEALME

WLKTRPILSPLTKGILGFVFTLTVPSERGLQRRRFVQNALNGNGDPNNMDRAVKLYKK

LKREITFHGAKEVSLSYSTGALASCMGLIYNRMGTVTTEAAFGLVCATCEQIADSQHR

SHRQMATTTNPLIRHENRMVLASTTAKAMEQMAGSSEQAAEAMEVANQTRQMVHAMRT

IGTHPSSSAGLKDDLLENLQAYQKRMGVQMQRFK"

ORIGIN

1 atgagtcttc taaccgaggt cgaaacgtac gttctttcta tcatcccgtc aggccccctc

61 aaagccgaga tcgcgcagag actggaaagt gtctttgcag gaaagaacac agatcttgag

121 gctctcatgg aatggctaaa gacaagacca atcttgtcac ctctgactaa gggaatttta

181 ggatttgtgt tcacgctcac cgtgcccagt gagcgaggac tgcagcgtag acgctttgtc

241 caaaatgccc taaatgggaa tggggacccg aacaacatgg atagagcagt taaactatac

301 aagaagctca aaagagaaat aacgttccat ggggccaagg aggtgtcact aagctattca

361 actggtgcac ttgccagttg catgggcctc atatacaaca ggatgggaac agtgaccaca

421 gaagctgctt ttggtctagt gtgtgccact tgtgaacaga ttgctgattc acagcatcgg

481 tctcacagac aaatggctac taccaccaat ccactaatca ggcatgaaaa cagaatggtg

541 ctggctagca ctacggcaaa ggctatggaa cagatggctg gatcgagtga acaggcagca

601 gaggccatgg aggttgctaa tcagactagg cagatggtac atgcaatgag aactattggg

661 acccatccta gctccagtgc tggtctgaaa gatgaccttc ttgaaaattt gcaggcctac

721 cagaagcgaa tgggagtgca gatgcagcga ttcaagtgat cctctcgtca ttgcagcaaa

781 tatcattggg atcttgcacc tgatattgtg gattactgat cgtctttttt tcaaatgtat

841 ttatcgtcgc tttaaatacg gtttgaaaag agggccttct acggaaggag tgcccgagtc

901 catgagggaa gaatatcaac aggaacagca tagtgctgtg gatgttgacg atggtc

//

**NS- GenBank: JX482560.1**

Influenza A virus (A/swine/Nigeria/12VIR4047-09/2011(H1N1)) segment 8 nuclear export protein (NEP) gene, partial cds; and nonstructural protein 1 (NS1) gene, complete cds

GenBank: JX482560.1

FASTA Graphics

Go to:

LOCUS JX482560 813 bp cRNA linear VRL 04-SEP-2012

DEFINITION Influenza A virus (A/swine/Nigeria/12VIR4047-09/2011(H1N1)) segment

8 nuclear export protein (NEP) gene, partial cds; and nonstructural

protein 1 (NS1) gene, complete cds.

ACCESSION JX482560

VERSION JX482560.1

DBLINK BioProject: PRJNA37813

KEYWORDS .

SOURCE Influenza A virus (A/swine/Nigeria/12VIR4047-09/2011(H1N1))

ORGANISM Influenza A virus (A/swine/Nigeria/12VIR4047-09/2011(H1N1))

Viruses; ssRNA viruses; ssRNA negative-strand viruses;

Orthomyxoviridae; Influenzavirus A.

REFERENCE 1 (bases 1 to 813)

AUTHORS Meseko,C.A., Heidari,A., Monne,I., Fusaro,A., Cattoli,G.,

Odaibo,G.N., Bakarey,A.S., Olaleye,D.O. and Capua,I.

TITLE Molecular characterization of pandemic A/H1N1 2009 influenza virus

isolated in pigs in Lagos, Nigeria

JOURNAL Unpublished

REFERENCE 2 (bases 1 to 813)

AUTHORS Meseko,C.A., Heidari,A., Monne,I., Fusaro,A., Cattoli,G.,

Odaibo,G.N., Bakarey,A.S., Olaleye,D.O. and Capua,I.

TITLE Direct Submission

JOURNAL Submitted (13-AUG-2012) FAO/OIE Reference Laboratory for Newcastle

Disease and Avian Influenza, OIE Collaborating Center for Diseases

at the Human-Animal Interface, Istituto Zooprofilattico

Sperimentale delle Venezie, Viale dell'Universita 10, Legnaro,

Padova 35020, Italy

COMMENT ##Assembly-Data-START##

Sequencing Technology :: Sanger dideoxy sequencing

##Assembly-Data-END##

FEATURES Location/Qualifiers

source 1..813

/organism="Influenza A virus

(A/swine/Nigeria/12VIR4047-09/2011(H1N1))"

/mol_type="viral cRNA"

/strain="A/swine/Nigeria/12VIR4047-09/2011"

/serotype="H1N1"

/isolation_source="nasal swab"

/host="swine"

/db_xref="taxon:1217303"

/segment="8"

/country="Nigeria: Lagos"

/collection_date="27-May-2011"

/note="lineage: swl; passage details: E1"

gene 1..>813

/gene="NEP"

/gene_synonym="NS2"

CDS join(1..30,503..>813)

/gene="NEP"

/gene_synonym="NS2"

/note="nonstructural protein 2"

/codon_start=1

/product="nuclear export protein"

/protein_id="AFQ90313.1"

/translation="MDSNTMSSFQDILMRMSKMQLGSSSEDLKRMTTQFESLKIYRDS

LGETVMRMGDLHYLQSRNEKWREQLGQKFEEIRWLIEEMRHRLKTTENSFEQITFMQA

LQLLLEVEQEI"

gene 1..660

/gene="NS1"

CDS 1..660

/gene="NS1"

/codon_start=1

/product="nonstructural protein 1"

/protein_id="AFQ90312.1"

/translation="MDSNTMSSFQVDCFLWHIRKRFADNGLVDAPFLDRLRRDQKSLK

GRGNTLGLDIETATLVGKQIVEWILKEESSETLRMTIASVPTSRYLSDMTLEEMSRDC

FMLMPRKKMIGPLCVRLDQAVMEKNIVLKANFSVIFNRLETLILLRAFTEEGAIVGEI

SPLPSLPGHTYEDVKNAVGVLIGGLEENDNTVRVSENIQRFAWRNCDENGRPSLPPEQ

K"

ORIGIN

1 atggactcca acaccatgtc aagctttcag gtagactgtt tcctttggca tatccgcaag

61 cgatttgcag acaatggatt ggttgatgcc ccattccttg atcggctccg ccgagatcaa

121 aagtccttaa aaggaagagg caacaccctt ggcctcgata tcgaaacagc cactcttgtt

181 gggaaacaaa tcgtggaatg gatcttgaaa gaggaatcca gcgagacact tagaatgaca

241 attgcatctg tacctacttc gcgctacctt tctgacatga ccctcgagga aatgtcacga

301 gactgtttca tgctcatgcc taggaaaaag atgataggcc ctctttgcgt gcgattggac

361 caggcagtca tggaaaagaa catagtactg aaagcgaact tcagtgtaat ctttaaccga

421 ttagagacct tgatactact aagggctttc actgaggagg gagcaatagt tggagaaatt

481 tcaccattac cttctcttcc aggacatact tatgaggatg tcaaaaatgc agttggggtc

541 ctcatcggag gacttgaaga gaatgacaac acagttcgag tctctgaaaa tatacagaga

601 ttcgcttgga gaaactgtga tgagaatggg agaccttcac tacctccaga gcagaaatga

661 aaagtggcga gagcaattgg gacagaaatt tgaggaaata agatggttaa ttgaagaaat

721 gcggcacaga ttgaaaacga cagagaatag tttcgaacaa ataacattta tgcaagcctt

781 acaactactg cttgaagtag aacaagagat aag

//
